# NOGOB receptor–mediated RAS signaling pathway is a target for suppressing proliferating hemangioma

**DOI:** 10.1172/jci.insight.142299

**Published:** 2021-02-08

**Authors:** Wenquan Hu, Zhong Liu, Valerie Salato, Paula E. North, Joyce Bischoff, Suresh N. Kumar, Zhi Fang, Sujith Rajan, M. Mahmood Hussain, Qing R. Miao

**Affiliations:** 1 Department of Foundations of Medicine, New York University Long Island School of Medicine, Mineola, New York, USA.; 2 Division of Pediatric Surgery, Department of Surgery, and; 3 Division of Pediatric Pathology, Department of Pathology, Children’s Research Institute, Medical College of Wisconsin, Milwaukee, Wisconsin, USA.; 4 Vascular Biology Program and Department of Surgery, Children’s Hospital Boston, Harvard Medical School, Boston, Massachusetts, USA.

**Keywords:** Angiogenesis, Vascular Biology, Cell cycle, Endothelial cells, Signal transduction

## Abstract

Infantile hemangioma is a vascular tumor characterized by the rapid growth of disorganized blood vessels followed by slow spontaneous involution. The underlying molecular mechanisms that regulate hemangioma proliferation and involution still are not well elucidated. Our previous studies reported that NOGOB receptor (NGBR), a transmembrane protein, is required for the translocation of prenylated RAS from the cytosol to the plasma membrane and promotes RAS activation. Here, we show that NGBR was highly expressed in the proliferating phase of infantile hemangioma, but its expression decreased in the involuting phase, suggesting that NGBR may have been involved in regulating the growth of proliferating hemangioma. Moreover, we demonstrate that NGBR knockdown in hemangioma stem cells (HemSCs) attenuated growth factor–stimulated RAS activation and diminished the migration and proliferation of HemSCs, which is consistent with the effects of RAS knockdown in HemSCs. In vivo differentiation assay further shows that NGBR knockdown inhibited blood vessel formation and adipocyte differentiation of HemSCs in immunodeficient mice. Our data suggest that NGBR served as a RAS modulator in controlling the growth and differentiation of HemSCs.

## Graphical abstract

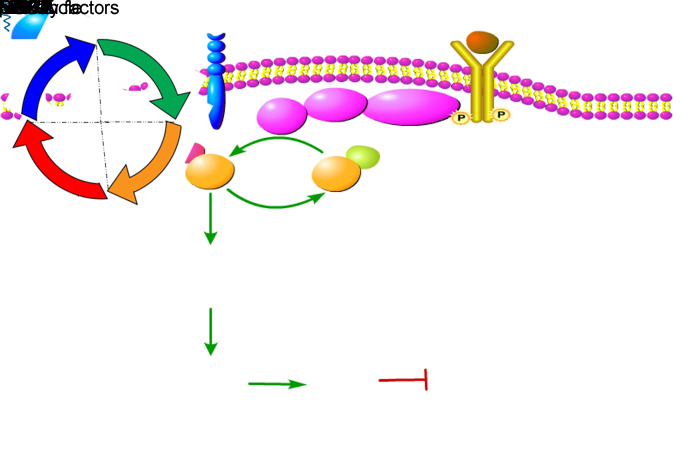

## Introduction

Infantile hemangioma, usually considered as a benign vascular tumor, exhibits a generally predictable life cycle that is divided into 3 stages (1–3). The proliferating phase spans the first year of postnatal life and is characterized by abundant immature endothelial cells without a defined vascular architecture. The involuting phase begins around 1 year of age and continues for an additional 3–5 years and is characterized by the presence of prominent endothelial-lined vascular channels. At the end of the involuting phase, the blood vessels are replaced by capillary-like vessels surrounded by loose fibrofatty tissue and represent the involuted phase (2, 4). The origin of hemangioma endothelial cells has been well studied (1, 5–8). The multipotent stem cells isolated from hemangioma tissues recapitulate hemangioma-like lesions in immunodeficient mice (9). However, the molecular mechanisms contributing to the development and progression of hemangioma remain to be elucidated (8, 10). Most infantile hemangioma does not require therapy and regresses spontaneously (11). Occasionally, 10%–15% of infantile hemangioma can cause significant cosmetic deformity or even induce life-threatening complications (12, 13). However, there is no uniformly safe and effective treatment for infants with hemangioma (14). Understanding the precise cellular mechanism that drives rapid growth and involution of hemangioma is essential for developing appropriate therapies.

Previous studies showed that the VEGFR signaling pathway plays an essential role in regulating hemangioma-associated blood vessel formation and maintenance (15, 16). Thus, VEGFR has been regarded as the most important target for treating hemangioma (17, 18). Tanyilidiz et al. reported that the serum basic FGF2 is higher in patients with hemangioma than in healthy control individuals, suggesting that FGF2 is an important growth factor for infantile hemangioma (19). In addition, a recent study by Zhang et al. revealed that EGF could significantly promote the in vitro proliferation and motility of hemangioma (20). The common feature of these growth factors is to activate RAS GTPase through respective RTKs (21, 22). RTKs are a family of cell surface receptors with an intracellular tyrosine kinase domain that promotes many cellular functions through the activation of RAS-dependent activation of AKT and ERK1/2 pathways (23). As summarized in the recent review (24, 25), many mutations primarily within the RAS pathway are involved in the pathogenesis of infantile hemangioma. However, the direct contribution of RAS to the pathogenesis of hemangioma has not been examined. Here, we demonstrate for the first time to our knowledge that RAS and NOGOB receptor (NGBR), a RAS modulator, were essential for growth and differentiation of infantile hemangioma.

As was shown in our recent paper (26), NGBR, a transmembrane protein, binds prenylated RAS and promotes the translocation of RAS from the cytosol to the plasma membrane, which is a critical step required for RTK-mediated activation of RAS. We demonstrated that NGBR is essential for vasculature development by promoting chemotaxis of endothelial cells and angiogenesis (27–30). In this study, we elucidate the roles of NGBR as a RAS modulator in regulating the proliferation and migration of hemangioma stem cells (HemSCs) in vitro as well as the differentiation of HemSCs in vivo. Our data illustrate that NGBR-mediated RAS activation played a critical role in HemSCs malignancy.

## Results

### NGBR was highly expressed in proliferating phase of infantile hemangioma.

**Figure 1:**
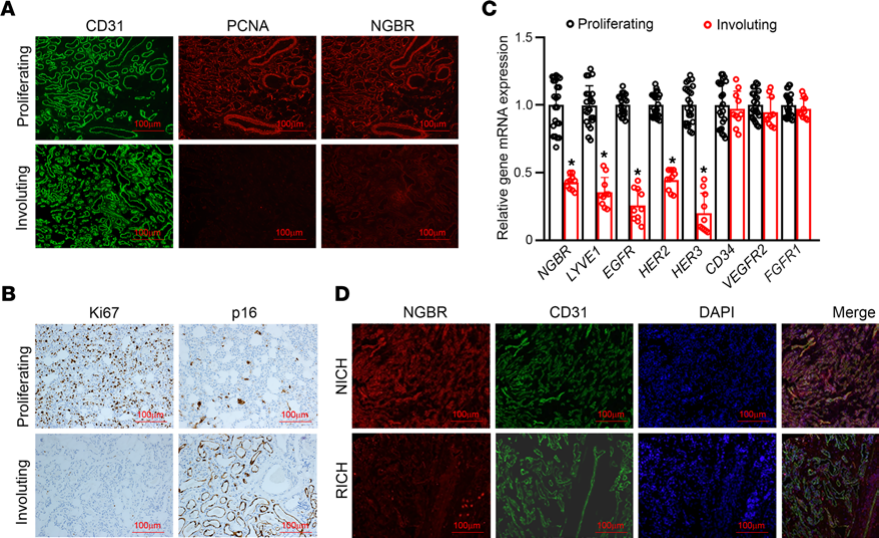
The expression of NGBR in proliferating and involuting phases of infantile hemangioma. (**A**) The IF staining of PCNA and NGBR increased in proliferating phase hemangioma tissues but decreased in involuting phase hemangioma tissues. Scale bar: 100 μm. (**B**) The IHC staining of Ki67 increased in proliferating phase hemangioma tissues, and the IHC staining of p16 increased in involuting phase hemangioma tissues. Scale bar: 100 μm. (**C**) The mRNA levels of *NGBR*, *LYVE1*, *EGFR*, *HER2*, *HER3, CD34*, *VEGFR2,* and *FGFR1* were detected by real-time reverse-transcription PCR in hemangioma tissues. *P < 0.05, involuting phase hemangioma tissues (*n* = 10) vs. proliferating phase hemangioma phase (*n* = 23). (**D**) The intense IF staining of NGBR is appreciated in NICH but not in RICH. Scale bar: 100 μm. Statistical analyses: 2-tailed unpaired Student’s *t* test (**C**); data are expressed as mean ± SEM. NGBR, NOGOB receptor; IF, immunofluorescence; PCNA, proliferating cell nuclear antigen; NICH, noninvoluting congenital hemangioma; RICH, rapidly involuting congenital hemangioma.

We first performed IHC to examine the expression of NGBR in proliferating and involuting infantile hemangioma obtained from patients. As shown in Figure 1A, CD31-positive capillaries and large blood vessels were present in both hemangiomas. In contrast, proliferating cell nuclear antigen (PCNA) was mainly expressed in proliferating hemangiomas. Similar to PCNA, NGBR was mainly expressed in proliferating hemangiomas (Figure 1A, right). Consistently, IHC staining of proliferating marker Ki67 was mainly expressed in proliferating hemangioma (Figure 1B, left). In contrast, IHC staining of p16, a senescence marker, was mainly detected in involuting hemangioma (Figure 1B, right). *NGBR* expression was significantly higher in proliferating hemangiomas than involuting hemangiomas (Figure 1C). As expected, we also found that lymphatic endothelial hyaluronan receptor-1 (*LYVE1*) and RTKs, such as *EGFR*, *HER2*, and *HER3*, were highly expressed in proliferating hemangiomas (Figure 1C). These results show that NGBR was highly expressed in proliferating hemangiomas. Involuting hemangiomas are subdivided into rapidly involuting congenital hemangiomas (RICHs) and noninvoluting congenital hemangiomas (NICHs) (31). Our studies show that NGBR expression was highly expressed only in NICH (Figure 1D). Thus, NGBR was highly expressed in proliferating hemangiomas and NICHs but not in involuting and RICHs.

GLUT1 is a well-defined biomarker for hemangioma vasculature (9, 32). To verify that NGBR highly expressed in hemangioma blood vessels, we performed coimmunofluorescence staining using antibodies specific for human GLUT1, CD31, smooth muscle actin (SMA), and caldesmon. Immunostaining sections were examined using confocal microscopy. As shown in the Supplemental Figure 1A (supplemental material available online with this article; https://doi.org/10.1172/jci.insight.142299DS1), high expression of NGBR in proliferating hemangioma was colocalized with both GLUT1- and CD31-positive endothelial cells of capillaries. In addition, NGBR was highly expressed in the SMA- and caldesmon-positive smooth muscle cells (Supplemental Figure 1, B and C) but weakly in GLUT1-negative endothelial cells of larger blood vessels (Supplemental Figure 1B), which are not hemangioma blood vessels. These results provide additional evidence for the high expression of NGBR in proliferating hemangioma cells.

### Knockdown of NGBR inhibited proliferation of HemSCs by cell cycle arrest.

**Figure 2:**
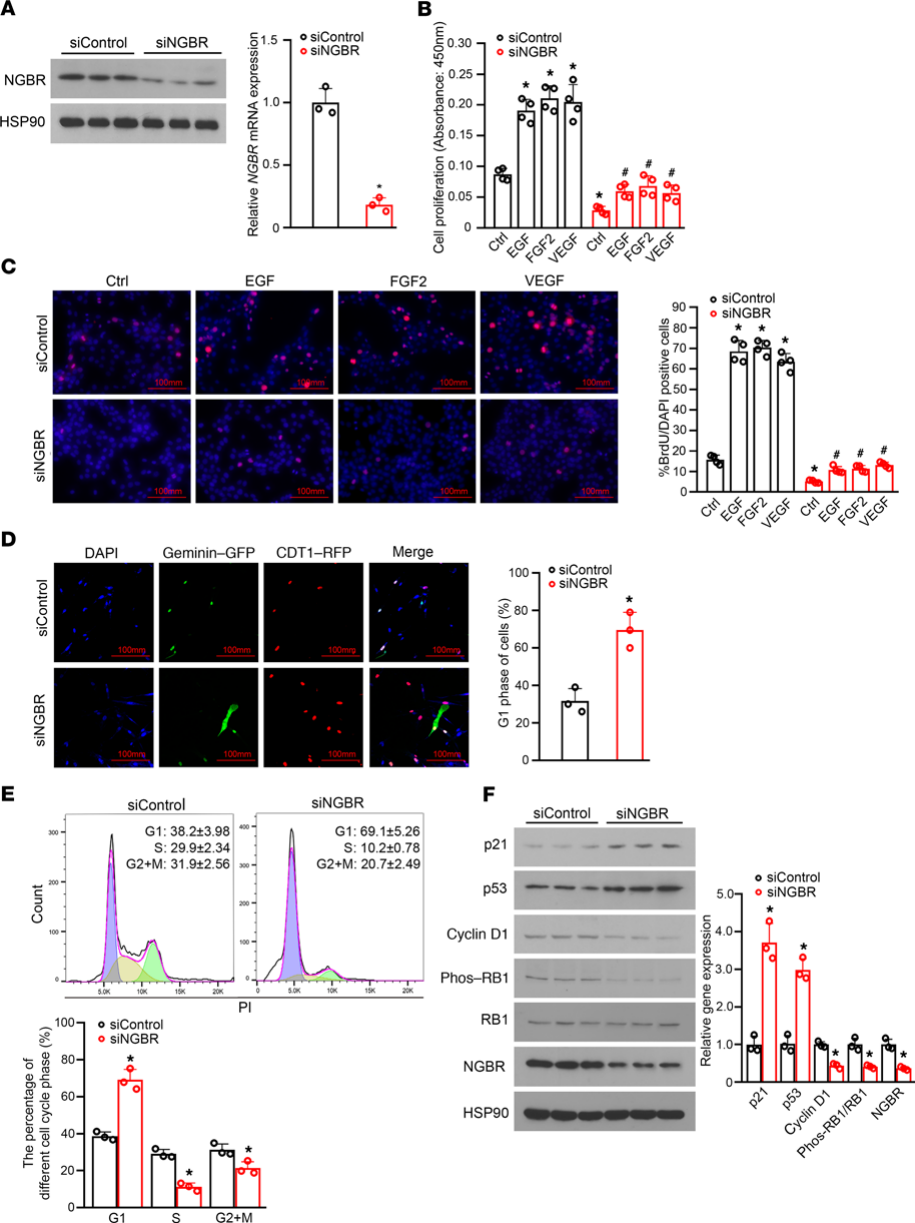
NGBR knockdown inhibits the proliferation of HemSCs in vitro. (**A**) NGBR knockdown was characterized by Western blot and RT-PCR. *P < 0.05 vs. control (siControl) cells (*n* = 3). (**B**) Cell proliferation modulated by NGBR deficiency was evaluated by cell proliferation assay after treatment with EGF, FGF2, and VEGF in HemSCs. The results were expressed as fold change relative to the initial cell number. *P < 0.05 vs. control (siControl) cells. ^#^*P* < 0.05 vs. control (siControl) cells treated with EGF, FGF2, and VEGF (*n* = 4), 3 repeats. (**C**) NGBR knockdown inhibited BrdU incorporation in HemSCs treated with growth factors. Cells treated with EGF, FGF2, and VEGF displayed more BrdU-positive cells than control groups. NGBR knockdown abolished EGF-induced, FGF2-induced, and VEGF-induced BrdU incorporation. Quantitative analysis of BrdU-positive cells was determined by ImageJ software. *P < 0.05 vs. control (siControl) cells. ^#^*P* < 0.05 vs. control (siControl) cells treated with EGF, FGF2, and VEGF (*n* = 4), 3 repeats. (**D**) HemSCs expressing FUCCI cell cycle markers were transiently transfected with siControl or siNGBR. After 24 hours, the cells were fixed, and the images were taken with a confocal microscope. Quantitative analysis of G_1_ phase cells was determined by ImageJ software. *P < 0.05 vs. control (siControl) cells (*n* = 3), 3 repeats. (**E**) HemSCs transfected with siNGBR underwent G_1_ phase arrest. The cell cycle distribution was analyzed by flow cytometry using PI staining. The percentage of different cell cycle phases presented in histogram form has been summarized in the bar graph (right). *P < 0.05 vs. control (siControl) cells (*n* = 3), 3 repeats. (**F**) Protein levels of NGBR, p21, p53, cyclin D1, RB1, and phosphorylated RB1 in HemSCs treated with siControl and siNGBR were determined by Western blot. *P < 0.05 vs. control (siControl) cells (*n* = 3). Statistical analyses: 2-tailed unpaired Student’s *t* test (**A**, **D**, **E**, and **F**) and 1-way ANOVA with Bonferroni’s post hoc test (**B** and **C**); data are expressed as mean ± SEM. HemSCs, hemangioma stem cells; NGBR, NOGOB receptor; siControl, control siRNA; siNGBR, NGBR siRNA; FUCCI, fluorescence ubiquitination cell cycle indicator; RB1, retinoblastoma; PI, propidium iodide.

As shown in a previous publication (9), HemSCs isolated from hemangioma tissues recapitulate hemangioma-like lesions in immunodeficient mice. We further used HemSCs to determine the cellular functions of NGBR, which is highly expressed in the proliferating hemangioma cells. We performed loss-of-function experiments using NGBR siRNA (siNGBR) that has been validated in our previous publication (33). The efficacy of NGBR knockdown in HemSCs was more than 80% as confirmed by Western blot and quantitative RT-PCR approaches (Figure 2A). After NGBR knockdown in HemSCs, we examined cell proliferation by BrdU incorporation assay. The proliferation of HemSCs was dramatically decreased in NGBR knockdown cells cultured in growth medium as well as in serum-free basal medium containing different RTK growth factors, such as VEGF, FGF2, and EGF (Figure 2B). This result was substantiated further using BrdU immunostaining (Figure 2C). As illustrated in Figure 2C, the percentage of cells that incorporated BrdU was significantly decreased in NGBR-silenced HemSCs, and NGBR deficiency also diminished growth factor–induced BrdU incorporation, suggesting that NGBR may be critical for the proliferation of HemSCs. We also performed the immunofluorescence (IF) staining of Ki67, a well-defined cell proliferative marker. As shown in the Supplemental Figure 2, NGBR depletion significantly decreased the number of Ki67-positive cells that were induced by RTK growth factors. Taken together, NGBR highly expressed in proliferating hemangioma contributes to the proliferation of HemSCs, and silencing of NGBR significantly inhibits the proliferative potential of hemangioma.

**Figure 3:**
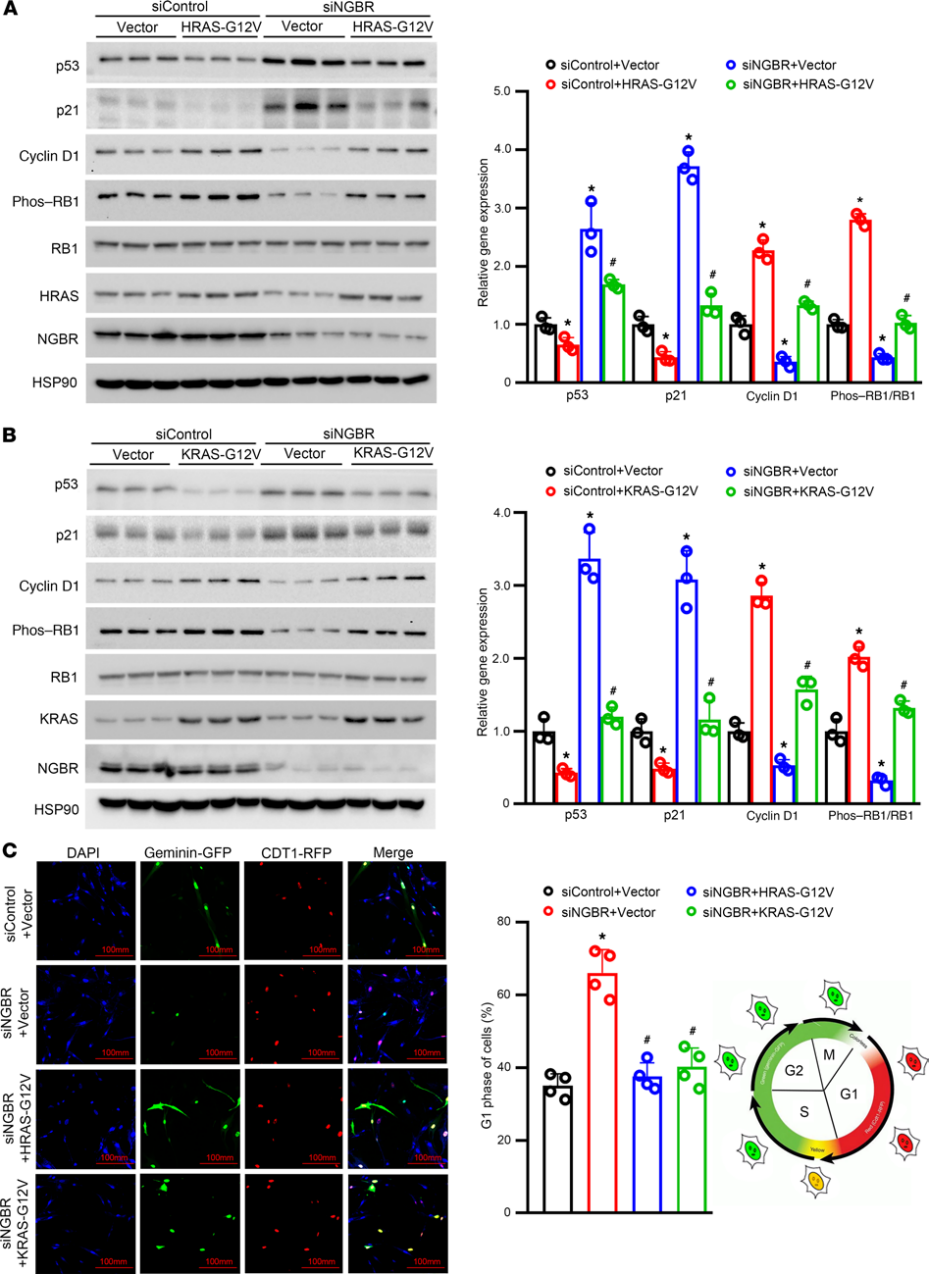
Overexpression of constitutively activated HRAS/KRAS diminishes NGBR deficiency caused G_1_ phase arrest. (**A** and **B**) siControl and siNGBR cells were transiently transfected with empty vector or HRAS-G12V/KRAS-G12V expression vector for 24 hours. Protein levels of NGBR, p21, p53, cyclin D1, RB1, and phosphorylated RB1 were determined by Western blot. Quantitative analysis of proteins was carried out using ImageJ software. *P < 0.05 vs. control (siControl) cells. ^#^*P* < 0.05 vs. siNGBR cells (*n* = 3). (**C**) siControl and siNGBR cells expressing the FUCCI fluorescent CDT1-RFP (red) and geminin-GFP (green) were transiently transfected with HRAS-G12V or KRAS-G12V expression vector for 24 hours. The images were taken with a confocal microscope. Quantitative analysis of G_1_ phase cells was determined by ImageJ software. *P < 0.05 vs. control (siControl) cells. ^#^*P* < 0.05 vs. siNGBR cells (*n* = 4), 3 repeats. Statistical analyses: 1-way ANOVA with Bonferroni’s post hoc test; data are expressed as mean ± SEM. NGBR, NOGOB receptor; siControl, control siRNA; siNGBR, NGBR siRNA; RB1, retinoblastoma; FUCCI, fluorescence ubiquitination cell cycle indicator.

To further explore the underlying mechanism by which NGBR regulates cell proliferation, we also examined the effects of NGBR knockdown on cell cycle using both the fluorescence ubiquitination cell cycle indicator (FUCCI) cell cycle assay and the flow cytometric assay. FUCCI is a sensor that employs RFP-fused CDT1 and GFP-fused geminin, which effectively labels individual G_1_ phase nuclei red and those in S/G_2_/M phases green, respectively (34). The FUCCI Cell Cycle Sensor allows visualization of cell cycle progression in live cells by exhibiting a dynamic color change from red to green in a single cell (35). As shown in Figure 2D, the percentage of G_1_ phase (red color) cells was significantly increased in HemSCs transfected with siNGBR compared with HemSCs treated with control siRNA (siControl). The results of the FUCCI cell cycle analysis demonstrate that the silencing of NGBR caused the G_1_ phase arrest of HemSCs. This result was substantiated further using the traditional flow cytometer assay. Consistently, NGBR knockdown dramatically increased the percentage of the G_1_ phase from 38.2% to 69.1% and reduced the percentage of S phase cells from 29.9% to 10.2% (Figure 2E), which implies that the silencing of NGBR in HemSCs blocked the transition of G_1_/S phase of the cell cycle. Cyclin D1 is required for the progression of the vertebrate cells preparing for the S phase during the G_1_ phase. Cyclin D1 regulates DNA synthesis to prepare for cell division, whereas p21, a p53 target gene, and retinoblastoma (RB1) play negative regulatory roles in this process (36, 37). Western blot results (Figure 2F) reveal that NGBR depletion significantly reduced the protein level of cyclin D1 and phosphorylation of RB1 (inactivation status) and resulted in the upregulated protein levels of p21 and p53 in HemSCs. Similar results were observed when either HRAS or KRAS were knocked down in HemSCs. The efficacy of siRNA knocking down HRAS and KRAS is shown in the Supplemental Figure 3A. As shown in Supplemental Figure 3B, upon treatment with either HRAS- or KRAS-specific siRNA, the G_1_ phase distribution increased from 36.5% to 67.7% and 66.6%, and the percentage of S phase decreased from 34.6% to 17.7% and 19.8%, respectively. Western blot results (Supplemental Figure 3C) show that knockdown of either HRAS or KRAS in HemSCs also decreased the protein levels of cyclin D1 and phosphorylation of RB1 but increased the protein levels of p21 and p53. To confirm the specific requirements of HRAS and KRAS in the G_1_ phase arrest of NGBR silencing HemSCs, we overexpressed either constitutively activated HRAS (HRAS-G12V) or KRAS (KRAS-G12V), respectively. As shown in Figure 3, A and B, overexpression of constitutively activated HRAS or KRAS in HemSCs treated with siNGBR restored the protein levels of cyclin D1 and phosphorylation of RB1, p53, and p21 to the levels shown in control HemSCs transfected with siControl and empty vector. Consequently, overexpression of constitutively activated HRAS or KRAS abolished the G_1_ phase arrest caused by NGBR silencing (Figure 3C). In summary, our data indicate that the NGBR-mediated RAS signaling pathway was required for preserving cyclin D1 expression and phosphorylation of RB1 to promote the transition of the G_1_ phase to S phase during the cell cycle of HemSCs.

### Silencing of NGBR decreased HemSCs migration.

**Figure 4:**
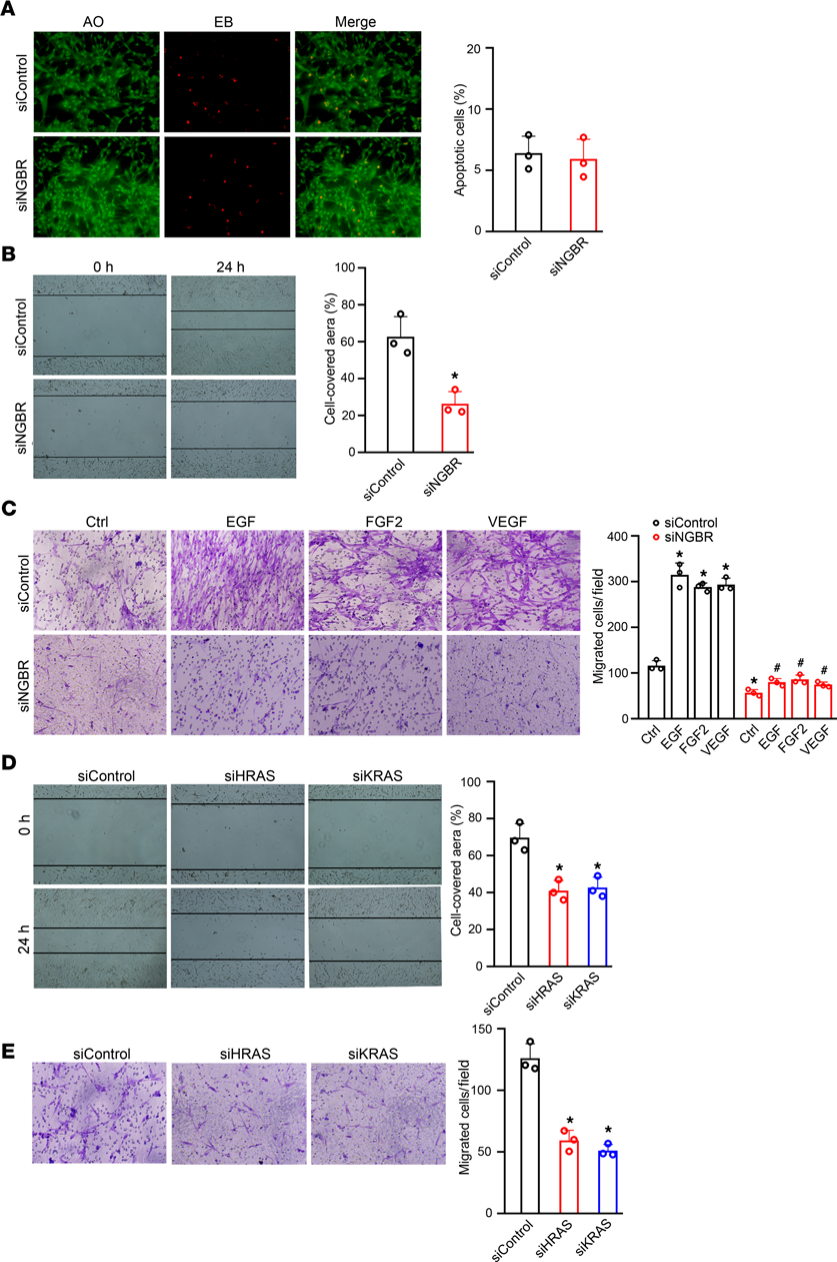
NGBR knockdown decreases the migration of HemSCs in vitro. (**A**) NGBR knockdown did not affect the cell viability of HemSCs. AO/EB staining was used for determining apoptotic HemSCs after treatment with siControl and siNGBR. The quantitative results show the average percentage of apoptotic cells (*n* = 3), 3 repeats. (**B**) The representative wound-healing images of HemSCs transfected with siControl or ​siNGBR at indicated times. Each condition was photographed in 4 separate fields. The quantitative results are presented as a bar graph (right). *P < 0.05 vs. control (siControl) cells (*n* = 3), 3 repeats. (**C**) Transwell migration assay of HemSCs transfected with either siControl or ​siNGBR with/without EGF, FGF2, and VEGF stimulation. The representative images of migrated cells are shown in the left panel. The bar graph in the right panel shows the quantitative numbers of migratory cells evaluated by ImageJ software. *P < 0.05 vs. control (siControl) cells. ^#^*P* < 0.05 vs. control (siControl) cells treated with EGF, FGF2, and VEGF (*n* = 3), 3 repeats. (**D**) Scratch wound healing assay shows reduced migration of either HRAS- or KRAS-deficient HemSCs. HemSCs transfected with siControl, siHRAS, or siKRAS were subjected to scratch wound healing assay. The representative images of wound healing are shown at indicated times. The bar graph in the right panel represents the percentage of the cell-covered area determined from each time point. *P < 0.05 vs. control (siControl) cells (*n* = 3), 3 repeats. (**E**) Transwell migration assay of HemSCs transfected with siControl, siHRAS, or siKRAS. Cell migration was determined using Corning Transwell chambers. The results presented are an average of migrated cell numbers in 4 random microscopic fields from 3 independent experiments. *P < 0.05 vs. control (siControl) cells (*n* = 3). Statistical analyses: 2-tailed unpaired Student’s *t* test (**A**, **B**, **D**, and **E**) and 1-way ANOVA with Bonferroni’s post hoc test (**C**); data are expressed as mean ± SEM. NGBR, NOGOB receptor; HemSCs, hemangioma stem cells; AO/EB, acridine orange/ethidium bromide; siControl, control siRNA; siNGBR, NGBR siRNA.

To evaluate the contribution of cell cycle arrest to the cell viability, we determined the effect of NGBR silencing on cell viability using acridine orange/ethidium bromide (AO/EB) staining. As shown in Figure 4A, NGBR knockdown did not increase the number of apoptotic cells, which show red EB staining in Figure 4A. To determine the roles of NGBR in regulating the motility and chemotaxis of HemSCs, we performed wound healing and Transwell migration assays, respectively. As shown in the wound healing assay (Figure 4B), we observed much less NGBR knockdown in HemSCs migrating into the wound area than control cells treated with nonsilencing siControl. Consistently, the results of Transwell migration assays show that NGBR knockdown also decreased the chemotaxis of HemSCs (Figure 4C). Similar results were observed for the silencing of either HRAS or KRAS in HemSCs (Figure 4, D and E). We further examined whether NGBR was also necessary for RTK growth factor–stimulated cell migration. As shown in Figure 4C, RTK growth factors EGF, FGF2, and VEGF induced the migration of HemSCs. The silencing of NGBR abolished RTK growth factor–stimulated migration of HemSCs. These results suggest that NGBR also was required for RTK growth factor–induced migration of HemSCs. Mesenchymal features have been identified in infantile hemangioma (38–40). However, NGBR knockdown did not change the expression of mesenchymal markers (vimentin and N-cadherin) and epithelial marker (E-cadherin) (Supplemental Figure 4). It indicates that NGBR may be not involved in regulating the epithelial to mesenchymal transition of HemSCs.

### NGBR knockdown decreased plasma membrane–associated RAS and diminished activation of RTK-mediated signaling pathway.

**Figure 5:**
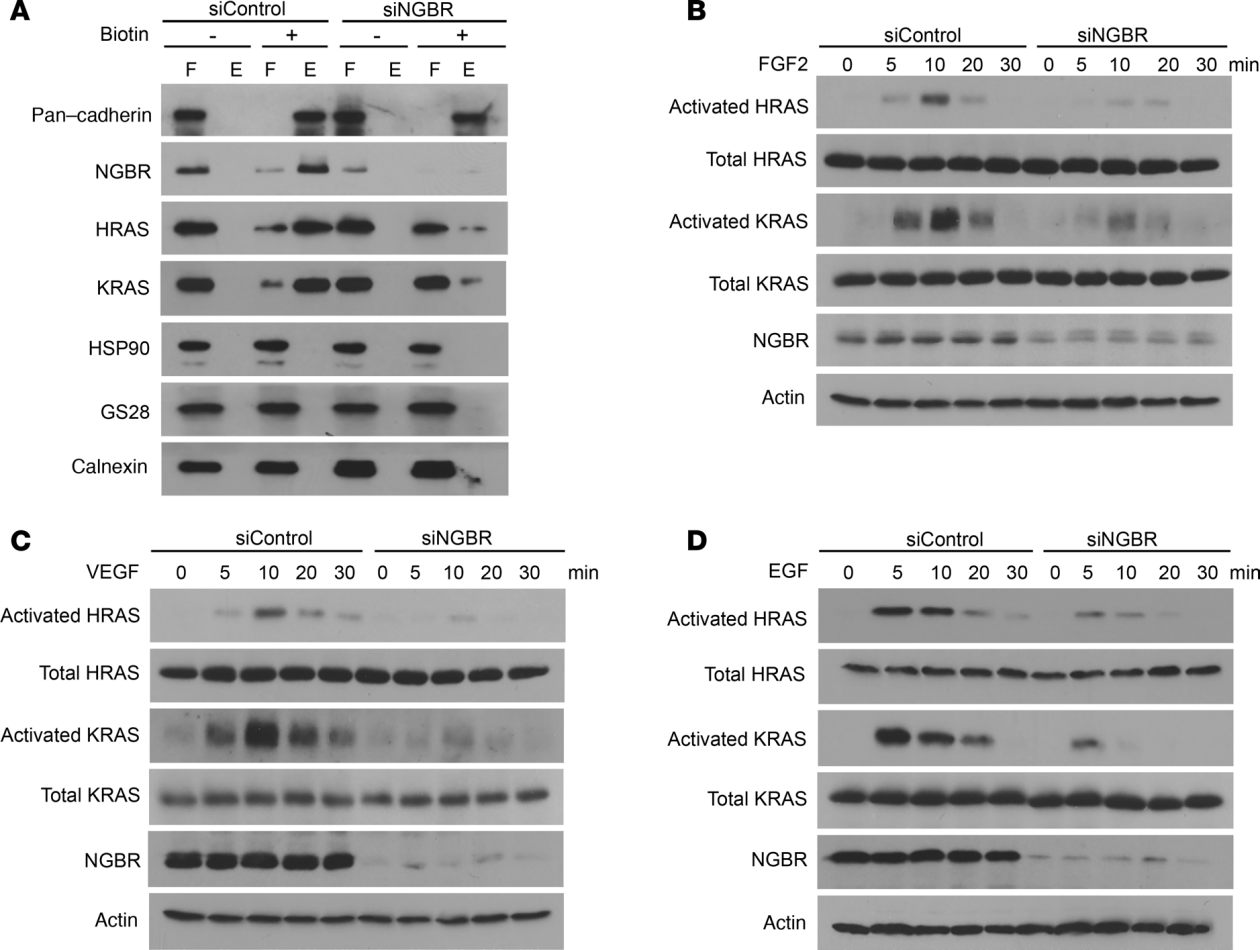
NGBR depletion attenuates RAS membrane accumulation and growth factor–stimulated RAS activation. (**A**) NGBR depletion decreased the protein levels of NGBR, HRAS, and KRAS in the fraction of biotinylated cell surface proteins. HemSCs surface proteins were biotinylated under nonpermeabilized conditions and isolated using streptavidin agarose resin from the Pierce Cell Surface Protein Isolation Kit as described in Methods. Proteins were determined by Western blot analysis using antibodies that detect endogenous proteins. Pan-cadherin, calnexin, and GS28 are markers of plasma membrane, ER membrane, and Golgi membrane markers, respectively. The plus symbol (+) denotes results for cells treated with the Sulfo-NHS-SS-Biotin reagent; the minus symbol (−) denotes results for cells that were not treated with the biotin reagent but were otherwise used in the kit procedure. The lanes designated “F” show proteins that flowed through the columns because they did not bind the avidin agarose resin, and the lanes designated “E” show proteins that were eluted from the columns after binding to the avidin agarose resin. (**B–D**) NGBR knockdown decreased the FGF2-induced (**B**), VEGF-induced (**C**), and EGF-induced (**D**) activation of HRAS and KRAS in HemSCs. The activated RAS proteins were isolated using GST-RBD beads, and protein levels were determined by Western blot. Data are validated in 3 independent experiments. NGBR, NOGOB receptor; HemSCs, hemangioma stem cells; GST-RBD, GST-tagged Ras-binding domain.

Our previous study showed that NGBR binds farnesylated RAS and promotes the translocation of RAS from the cytosol to the plasma membrane, which is a critical step in activating RTK signaling pathways (26). To further investigate the role of NGBR-mediated RAS recruitment and activation of the RTK signaling pathway in hemangioma, we first examined the effect of NGBR knockdown on RAS localization in the plasma membrane of HemSCs. We isolated cell surface proteins using the biotinylation approach as described in Methods and our previous publication (26). As shown in Figure 5A, NGBR was detected in the cell surface protein fraction. The purity of the isolated cell surface protein was confirmed by being free from contamination of Golgi (GS28) and endoplasmic reticulum (calnexin) proteins. However, the amount of NGBR diminished in the cell surface protein fractions of HemSCs transfected with siNGBR. Consistent with our previous report (26), NGBR knockdown in HemSCs also decreased the levels of membrane-associated HRAS and KRAS (Figure 5A), although the protein levels of HRAS and KRAS in total cell lysates did not change (Figure 5B). However, NGBR knockdown did not affect the level of the other plasma membrane–associated protein, pan-cadherin (Figure 5A). This suggests that NGBR was specifically required for the plasma membrane localization of RAS in HemSCs.

**Figure 6:**
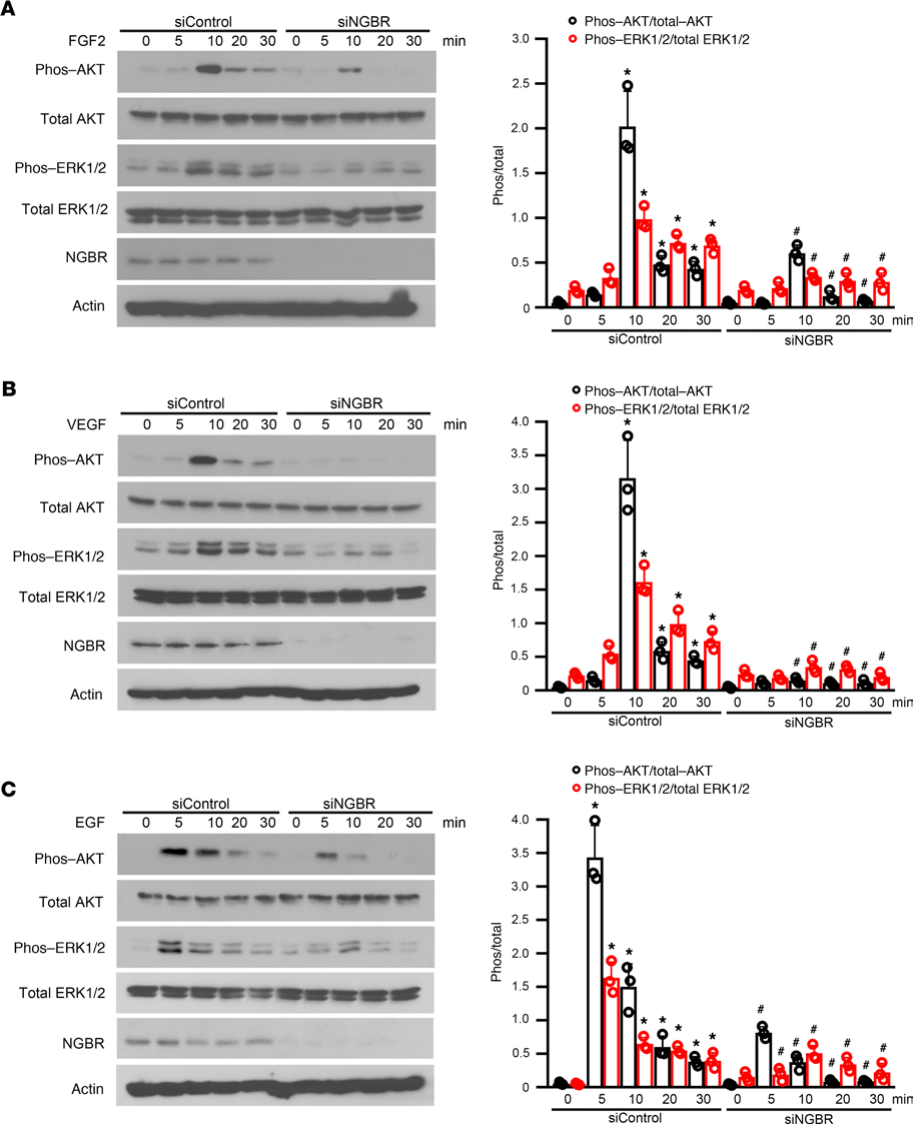
NGBR knockdown attenuates growth factor–induced phosphorylation of AKT and ERK1/2 in HemSCs. Protein levels were determined by Western blot. NGBR knockdown reduced the FGF2-induced (**A**), VEGF-induced (**B**), and EGF-induced (**C**) phosphorylation of AKT and ERK1/2 in HemSCs. Twenty-four hours after siControl or siNGBR transfection, cells were arrested overnight in serum-free medium and then stimulated with FGF2 (100 ng/mL), VEGF (100 ng/mL), and EGF (100 ng/mL) in serum-free medium at indicated times (5, 10, 20, and 30 minutes). The growth factor–induced phosphorylation AKT and ERK1/2 were determined by Western blot. Total AKT, total ERK1/2, and actin protein levels were used as respective loading controls. Quantitative analysis of phosphorylated proteins was carried out using ImageJ software, and proteins were normalized to total proteins correspondingly. *P < 0.05 vs. control (siControl) cells. ^#^*P* < 0.05 vs. control (siControl) cells treated by FGF2, VEGF, and EGF (*n* = 3), 3 repeats. Statistical analyses: 1-way ANOVA with Bonferroni’s post hoc test; data are expressed as mean ± SEM. NGBR, NOGOB receptor; HemSCs, hemangioma stem cells; siControl, control siRNA; siNGBR, NGBR siRNA.

To elucidate the roles of NGBR in regulating RTK-mediated RAS activation, we treated HemSCs in serum-free basal medium with different RTK growth factors, such as FGF2, VEGF, and EGF, for different time periods and used glutathione-tagged (GST-tagged) RAS-binding domain (RBD) of RAF1 to pull down activated RAS as described in our previous publication (26). As shown in Figure 5, B–D, RTK growth factors, such as FGF2, VEGF, and EGF, induced the activation of both HRAS and KRAS in HemSCs. However, NGBR knockdown attenuated RTK growth factor–stimulated RAS activation. These results suggest that NGBR-mediated RAS recruitment was required for RTK-mediated RAS activation in HemSCs. To further evaluate the involvement of NGBR in regulating RTK-mediated signaling, we examined RAS downstream signaling and the phosphorylation of AKT, ERK1/2, and p38MAPK. As shown in Figure 6, treatment with RTK growth factors increased the phosphorylation of both AKT and ERK1/2 in control HemSCs transfected with nonsilencing siControl. However, NGBR knockdown diminished RTK growth factor–stimulated phosphorylation of AKT and ERK1/2, although the silencing of NGBR did not affect the total protein levels of AKT and ERK1/2. In addition, RTK growth factors also stimulated the phosphorylation of p38MAPK, which was diminished in HemSCs transfected with siNGBR (Supplemental Figure 5).

### NGBR knockdown suppressed differentiation and blood vessel formation of HemSCs in vivo.

**Figure 7:**
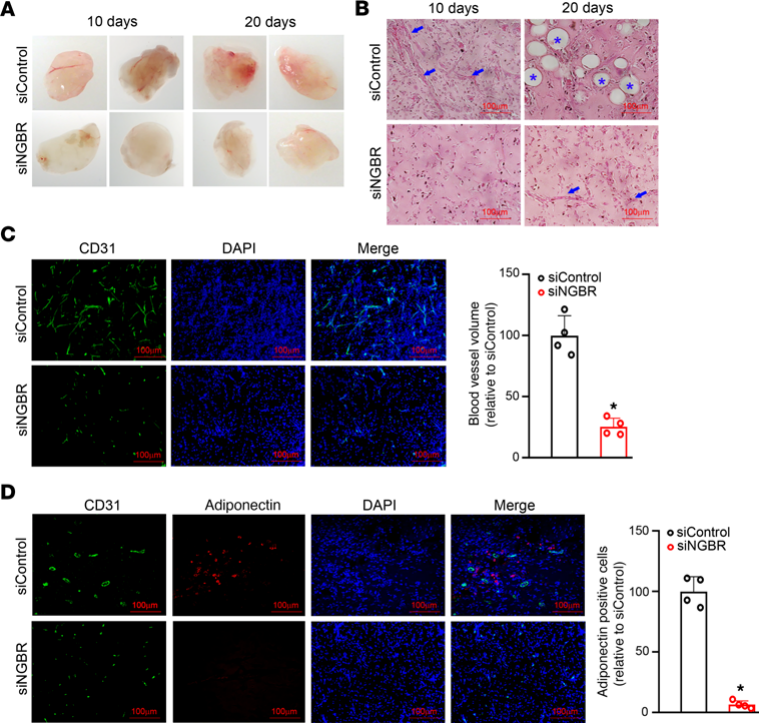
NGBR is required for the differentiation of HemSCs to blood vessels or adipocytes in vivo. (**A**) Representative images of implants isolated from the nude mice are shown in the left panel. Clonal siControl or siNGBR HemSCs were suspended in Matrigel and injected into nude mice. The implants were collected at indicated time points (day 10 and day 20). NGBR knockdown reduced the angiogenesis and adipogenesis in the implants of HemSCs. (**B**) H&E staining of siControl and siNGBR HemSCs implants at the corresponding time points. Clonal HemSCs at passage 5 were used. Arrows point to the blood vessels, and stars point to the adipocytes. (**C**) IF staining of day 10 implants. IF staining of human CD31 (green) is shown on the left, followed by DAPI (blue) staining and a merged image. NGBR depletion decreased blood vessel formation on day 10. Quantitative analysis of positive CD31 staining was carried out using ImageJ software. *P < 0.05 vs. control (siControl) (*n* = 4). (**D**) IF staining of implants on day 20. IF staining of human CD31 (green) is shown on the left, followed by adiponectin (red) and DAPI (blue) staining, and a merged image. NGBR depletion decreased adipogenesis on day 20. Quantitative analysis of positive adiponectin staining was carried out using ImageJ software. *P < 0.05 vs. control (siControl) (*n* = 4). Scale bar: 100 μm. Statistical analyses: 2-tailed unpaired Student’s *t* test (**C** and **D**); data are expressed as mean ± SEM. NGBR, NOGOB receptor; HemSCs, hemangioma stem cells; siControl, control siRNA; siNGBR, NGBR siRNA.

To determine whether NGBR knockdown could suppress differentiation and blood vessel formation of HemSCs, we followed the protocol described in the previous publication (9) to carry out the Matrigel assay in vivo. Matrigel is a solubilized basement membrane protein preparation that supports cell growth and differentiation for in vitro and in vivo angiogenesis studies (9, 41). Normal control and NGBR-deficient HemSCs were resuspended in Matrigel and subcutaneously injected into 8-week-old athymic *nu/nu* mice (1.5 × 10^6^ cells/200 μl Matrigel/animal). Consistent with a previous report (9), both Matrigel morphology (Figure 7A) and H&E staining (Figure 7B) results show that blood vessel formation occurred in the Matrigel implanted with normal control HemSCs 10 days after implantation. After 20 days in vivo differentiation, blood vessels were still present, but large lipid-filled adipocytes became prominent. However, many fewer blood vessels and adipocytes existed in the Matrigel carrying NGBR-deficient HemSCs, which indicates that NGBR was required for in vivo differentiation of HemSCs to form blood vessels and adipocytes. To verify that the newly formed blood vessels and adipocytes were derived from human HemSCs, we performed IF staining using human-specific antibodies. Human CD31 antibody was used to detect blood vessels derived from HemSCs (Figure 7C), and human adiponectin and PPARγ antibodies were used to detect adipocytes derived from HemSCs (Figure 7D and Supplemental Figure 6A). The specificity of the anti-human CD31 and adiponectin antibodies was confirmed by negative staining in mouse tissues (Supplemental Figure 6B). After 10 days of in vivo differentiation, the volume of CD31-positive blood vessels in the Matrigel of NGBR-deficient HemSCs was only about 20%–30% of the amount of blood vessels generated from normal control HemSCs. After 20 days in vivo differentiation, lipid-filled adipocytes were barely found in the Matrigel implants of NGBR-deficient HemSCs. These results suggest that NGBR was required for the differentiation and blood vessel formation of HemSCs.

## Discussion

Hemangioma is a vascular tumor of infancy characterized by a proliferative rapid growth phase (42, 43). Many of the tumor signaling pathways are involved in the development of hemangioma, reflecting the malignancy of proliferating hemangioma (24, 25). As a RAS modulator (26), NGBR is highly expressed in proliferating hemangioma and required for promoting the proliferation and migration of HemSCs in vitro as well as the differentiation of HemSCs to form blood vessels and adipocytes in the Matrigel implanted into nude mice. Mechanistically, NGBR in HemSCs is essential for promoting the translocation of farnesylated RAS from the cytosol to the plasma membrane and RTK growth factor–stimulated activation of the RAS signaling pathways.

RAS is a common intermediate mediator of RTKs for activating downstream kinases, such as phosphatidylinositol-3-OH kinase (PI3K)/AKT and RAF1 kinase/ERK1/2 (23, 44). The aberrant activation of RAS signaling is associated with human vascular diseases, such as hematological malignancies and vascular disorders (45, 46). The proangiogenic effects of KRAS are attributed in part to the downregulation of thrombospondin, an endogenous inhibitor of angiogenesis (47). Studies have revealed a link between KRAS-mediated cellular transformation and the downregulation of thrombospondin-1 (TSP-1), which leads to increased angiogenesis (48). In the context of hemangioma, it has been shown that TSP-1 is downregulated in tissue biopsies of the proliferating phase. Such downregulation of TSP-1 was not observed in the involuting phase (48, 49). In addition, given the role of KRAS in regulating TSP-1 expression and the association of overexpressed TSP-1 with proliferating hemangioma, it is plausible that targeting the KRAS-mediated pathway might be beneficial. Therefore, understanding the underlying mechanism of RAS activation in hemangioma should help develop potential therapeutic approaches.

Many RTK growth factors, such as VEGF, PDGF, FGF2, and EGF, have been demonstrated in the pathogenesis of hemangioma. Many reports have confirmed that excessive VEGF expression in hemangioma tissue parallels the proliferating phase of its growth (50). Conversely, in the involuting phase, VEGF expression rapidly decreases. The abnormal activation of VEGFA and VEGFR2 on the cell surface endows robust capabilities of proliferation, colony formation, migration, and invasion (51, 52). In addition, the activation of the VEGF signaling pathway may also be beneficial to the survival of hemangioma endothelial cells during angiogenesis and vasculogenesis (53). Besides, VEGF also acts in an autocrine manner to promote RAS activation and tumor cell growth through the VEGF receptor neuropilin-1 (54). Walter et al. provided the first evidence for the regulatory role of PDGFB and PDGF receptor β (PDGFRβ) signaling in hemangioma. They established a genetic linkage with chromosome 5q in the 3 familial hemangiomas. They proved that PDGFB and PDGFRβ signaling plays a role in hemangioma pathogenesis (55). In addition, data from a separate study demonstrated that PDGFB and PDGFRβ signaling might act as an intrinsic negative regulator of hemangioma involution. They found that PDGFB is elevated during the proliferating phase and inhibits adipocyte differentiation (56). A previous report that showed higher serum FGF2 in patients with hemangioma than in healthy control individuals also suggested that FGF2 is a critical growth factor for infantile hemangioma (19). Zhang et al. revealed that EGF could significantly promote the in vitro proliferation and motility of hemangioma (20). In conclusion, these findings suggest that multiple RTK signaling pathways are involved in the proliferating hemangioma.

How to target the concurrent multiple RTK growth factor–stimulated cell proliferation should be the therapeutic challenges for proliferating hemangioma. Binding of an RTK ligand to their respective receptors stimulates the receptors’ intrinsic protein tyrosine kinase activity, which subsequently activates RAS in the plasma membrane and RAS-dependent signal transduction cascade requested for cell proliferation and migration (57). As a central player of the RTK-mediated signaling pathway, RAS should be a promising target for mitigating the concurrent RTK signaling in the proliferating hemangioma. However, RAS is a difficult, directly targeted protein because of its smooth surface conformation (58). Many efforts have been devoted to developing alternative approaches to targeting RAS signaling modulators, such as inhibiting RAS farnesylation (58).

In our opinion, NGBR is a potential RAS target because NGBR is required for the translocation of RAS from the cytosol to the plasma membrane. RAS plasma membrane localization is necessary to promote the activation of the RAS-mediated signaling pathway because the RAS activators and effectors are localized to the plasma membrane (59, 60). Our previous results indicated that the hydrophobic cytosolic domain of NGBR binds farnesylated RAS (26). We previously demonstrated that NGBR is essential for RAS plasma membrane translocation in tumor cells, and NGBR overexpression recapitulates the oncogenic functions of RAS in cell transformation and tumor growth (26, 33, 61–63). Here, we further elucidate the roles of NGBR in regulating malignancy of proliferating hemangioma. Based on the intensive expression of NGBR in proliferating infantile hemangioma and NICH, but not in involuting hemangioma and RICH, our favorite hypothesis is that highly expressed NGBR promoted the growth of proliferating hemangioma via NGBR-mediated RAS recruitment and RAS-dependent RTK signaling pathways (Supplemental Figure 7). As shown in Figure 5, Figure 6, and Supplemental Figure 5, many RTK growth factors promoted RAS activation in HemSCs, which is the required signaling for the malignancy of proliferating hemangioma. Data, as shown in Figure 5, Figure 6, and Supplemental Figure 5, also demonstrate that the depletion of NGBR in HemSCs blocked the translocation of HRAS and KRAS to the plasma membrane and diminished multiple RTK growth factor–induced receptor tyrosine kinase signaling pathways. Furthermore, we demonstrate that NGBR depletion could cause cell cycle arrest by increasing p21 and decreasing cyclin D1 and phosphorylation of RB1 (Figure 2). The functional assay results suggest that targeting NGBR was a feasible approach to suppress the RTK growth factor–stimulated proliferation and migration of HemSCs in vitro (Figure 2 and Figure 4) and decrease the differentiation and blood vessel formation of HemSCs in vivo (Figure 7). However, the molecular mechanism of regulating NGBR expression in proliferating and involuting hemangioma and the role of NGBR in regulating the differentiation of HemSCs to adipocytes need further investigation.

NOGOB is a member of the reticulon membrane protein family (64, 65), and its extracellular domain, a soluble form detected in circulation (66, 67), serves as a chemoattractant for endothelial cells (27, 66). NGBR was identified as a receptor specific for the soluble NOGOB-stimulated endothelial cell migration and blood vessel formation (27). As shown in Supplemental Figure 8, NGBR knockdown did not change the protein and mRNA levels of NOGOB in HemSCs, as determined by Western blot and real-time PCR, respectively (Supplemental Figure 8, A and B). NGBR knockdown also did not affect the levels of soluble NOGOB in the culture medium, as determined by ELISA (Supplemental Figure 8C). Unlike NGBR knockdown (Figure 2), NOGOB knockdown did not change the levels of phopsho-RB1, cyclin D1, p53, and p21 in HemSCs (Supplemental Figure 9A). Consequently, NOGOB knockdown did not affect cell proliferation, as determined by BrdU-based cell proliferation assay and Ki67 staining (Supplemental Figure 9, B and C). Similarly, NOGOB knockdown did not impair the migration of HemSCs, as determined by Transwell migration assay (Supplemental Figure 10A) and wound healing assay (Supplemental Figure 10B). These data suggest that NGBR-mediated proliferation and migration of HemSCs were not dependent on endogenous membrane-bound NOGOB. The differential functions of soluble and membrane-bound NOGOB in the context of hemangioma still need further investigation.

In summary, our study demonstrates that NGBR played a vital role in regulating the concurrent RTK growth factor–stimulated proliferation and migration of HemSCs. Our data demonstrate that NGBR-mediated RAS membrane accumulation and activation may have contributed to the malignancy of infantile hemangioma. Our findings suggest that NGBR was a promising therapeutic target for attenuating RAS signaling in infantile hemangioma induced by concurrent RTK growth factors, such as EGF, FGF2, and VEGF.

## Methods

### Reagents and antibodies.

HemSCs had been established in-house at Harvard Medical School (9). HemSCs were cultured in fibronectin-coated (1 μg/cm^2^) plates with EBM-2 medium (catalog CC-3156, Lonza) supplemented with 20% FBS as described previously (9). Deidentified specimens of hemangiomas were obtained from the Division of Pediatric Pathology at the Medical College of Wisconsin. Primary antibody human anti-rabbit antibodies against cyclin D1 (catalog 2978), p21 (catalog 2947), p53 (catalog 2527), phospho-RB1 (catalog 8516), RB1 (catalog 9313), HSP90 (catalog 4877), p44/42MAPK (catalog 9194), phospho-p44/42MAPK (catalog 8544), pan-cadherin (catalog 4068), p38MAPK (catalog 9212), phospho-p38MAPK (catalog 9211), AKT (catalog 4691), and phospho-AKT (catalog 9611) were all obtained from Cell Signaling Technology. The antibody against actin (catalog 66009) was purchased from Proteintech. Primary antibody against GS28 (catalog 611184) was purchased from BD Transduction Laboratories. The antibodies against HRAS (catalog GTX116041), KRAS (catalog GTX132480), vimentin (catalog GTX100619), E-cadherin (catalog GTX100443), N-cadherin (catalog GTX127345), and calnexin (catalog GTX109669) were all purchased from GeneTex. The primary anti-NOGOB antibody (catalog sc-271878) and NOGOB siRNA (catalog sc-43974) were purchased from Santa Cruz Biotechnology. The primary anti-NGBR antibody (catalog ab16835) was purchased from Abcam. The HRP-conjugated anti–rabbit (711-005-152) and anti–mouse (711-005-151) IgG secondary antibodies were purchased from Jackson ImmunoResearch Laboratory. Alexa Fluor 488/568–labeled anti-rabbit and anti-mouse IgG secondary antibodies were obtained from Thermo Fisher Scientific.

### IF.

IF staining was performed on 5 μm paraffin-embedded sections using respective antibodies from Thermo Fisher Scientific (GLUT1, catalog PA5-16793; Ki67 catalog 701198), BD Biosciences (PCNA, catalog 610664), MilliporeSigma (SMA, catalog A2547; caldesmon, catalog SAB4503188), Cell Signaling Technology (PPARγ, catalog 2435), and LifeSpan BioSciences (CD31, catalog LS-C286337; adiponectin, catalog LS-C337545). NGBR antibody used for IF staining has been described and characterized in our previous publication (68). After dewaxing and rehydration, the sections were sequentially covered with 3% H_2_O_2_ in methanol for 10 minutes to eliminate endogenous peroxidase activity. After washing with PBS, the sections were first incubated with blocking solution (Agilent DAKO) at 37°C for 30 minutes, then incubated with primary antibodies at 4°C overnight and subsequently incubated with Alexa Fluor secondary antibodies (Thermo Fisher Scientific). Cell nuclei were stained with DAPI, and the sections were observed using a fluorescence confocal microscope (Zeiss LSM510).

### Reverse-transcription quantitative PCR.

Total RNA was extracted from cells or human tissue samples using a RNeasy Kit (QIAGEN). The cDNA was reverse-transcribed from 2 μg total RNA using iScript cDNA Synthesis Kit (Bio-Rad) according to the manufacturer’s instructions. The quantitative PCR was run on the Bio-Rad MyiQ Real-Time PCR Detection System using iTaq Universal SYBR Green Supermix (Bio-Rad). The relative mRNA expression of each gene was normalized to the housekeeping gene (β-actin) mRNA levels. The real-time PCR primers were synthesized by Integrated DNA Technologies. Primer sequences can be found in Supplemental Table 1.

### Cell proliferation assay.

BrdU-based cell proliferation assay was carried out according to manufacturer’s instructions (Cell Signaling Technology). The cells were plated at a density of 1 × 10^4^ cells/well in 96-well plates. Cells were transfected with nonsilencing siControl and siNGBR overnight. Then, the cells were treated with RTK growth factors (100 ng/mL EGF, FGF2, or VEGF) for 24 hours in serum-free culture medium containing 1× BrdU solution. After incubation at room temperature for 30 minutes with fixing/denaturing solution, the fixing/denaturing solution was carefully removed, and 100 μl of 1× BrdU detection antibody solution was added into each well. After incubation at room temperature for 1 hour with gentle shaking, the BrdU antibody solution was removed, and the cells were washed twice with 300 μl 1× wash buffer. After incubation with 1× HRP-linked antibody solution at room temperature for 1 hour, 100 μl TMB substrate was added into each well for 5–30 minutes at room temperature to monitor the color development. After adding 100 μl Stop Solution into each well, the quantitative amount of incorporated BrdU was determined by measuring absorbance at 450 nm. Alternatively, BrdU-incorporated cells were visualized by incubating with fluorescence conjugated secondary antibody after the incubation with BrdU detection antibody solution.

### FUCCI cell cycle assay.

Cell cycle was evaluated using FUCCI Cell Cycle Sensor Kit (Thermo Fisher Scientific) (35). HemSCs expressing FUCCI fluorescent CDT1-RFP (red) and geminin-GFP (green) were transiently transfected with siRNA or plasmid DNA of HRAS-G12V/KRAS-G12V expression vector for 24 hours. Images of cells expressing either CDT1-RFP or geminin-GFP were taken with a Nikon Ti confocal microscope. The fluorescence intensities of RFP and GFP were quantified using ImageJ (NIH) software.

### Cell cycle analysis with flow cytometer.

For cell cycle analysis, cells were cultured in 60 mM culture dishes and transfected with siControl or siNGBR. Twenty-four hours after transfection, cells were harvested by trypsinization and fixed overnight with 70% cold ethanol at –20°C. The cells were subsequently centrifuged at 300*g* for 5 minutes and incubated with propidium iodide (PI) working solution (100 μg/mL PI and 100 μg/mL RNAse A) for 30 minutes at 37°C. BD LSRII flow cytometer was used to detect the cell cycle. FlowJo software was used to calculate the percentages of cells in each cycle phase.

### Western blot.

Cells were harvested and lysed in a lysis buffer containing 20 mM Tris-HCl, pH 7.5, 150 mM NaCl, 1 mM EDTA, 1 mM EGTA, 2.5 mM sodium pyrophosphate, 1 mM Na3VO4, 1 mM PMSF, 1% Triton X-100, and 1 μg/mL leupeptin. The protein concentration was determined using the BCA Protein Assay Kit (Bio-Rad). After separation using polyacrylamide gel electrophoresis, samples were then transferred to nitrocellulose blotting membrane (GE Healthcare Life Sciences) and incubated with primary-specific antibodies at 4°C overnight. The protein bands were developed using the ECL Western Blotting substrate (GE Healthcare Life Sciences) and were normalized to respective housekeeping proteins of HSP90 or actin. All Western blot experiments were repeated at least 3 times. See complete unedited blots in the supplemental material.

### Apoptosis assay with AO/EB staining.

The cells were cultured in 8-well chamber slides with EBM-2 medium supplemented with 20% FBS for 48 hours. After the indicated treatment with siControl or siNGBR, the cells were stained with AO (100 μg/mL) and EB (100 μg/mL), purchased from MilliporeSigma, and observed under a fluorescence confocal microscope (Zeiss LSM510). The normal and early apoptotic cells were stained with AO to display bright green fluorescence, whereas the late apoptotic cells were stained with EB to display orange fluorescence.

### Cell migration assay.

Cell migration was evaluated by wound healing assay and Transwell migration assay. HemSCs cells were seeded in 6-well-plates, and the cell monolayer was scratched with a sterile tip in the middle when cell confluence reached 80%. Then, the cell debris was washed away with culture medium. The pictures of cell morphology at different time points were taken with the Nikon Eclipse TS100 microscope. The narrowest distance of the gap between the front lines was measured by ImageJ software. Transwell migration assay was performed using the 8 μm pore size membrane chamber (Corning) in 24-well plates. Briefly, 2 × 10^5^ cells were plated in the upper chamber with EBM-2 medium supplemented with 0.5% FBS. For 6 hours, cells were allowed to migrate to the bottom chamber, which contained RTK growth factors (100 ng/mL EGF, FGF2, or VEGF) in EBM-2 medium supplemented with 0.5% FBS or full growth medium. After fixation with 4% paraformaldehyde and staining with 0.1% crystal violet and PBS, images of cells that migrated across the pore membrane were captured with Nikon Eclipse TS100 microscope.

### Isolation of plasma membrane proteins.

The Pierce Cell Surface Protein Isolation Kit (Thermo Fisher Scientific) was used for isolating plasma membrane proteins for Western blot analysis. In brief, the cells were first labeled with EZ-Link Sulfo-NHS-SS-Biotin for 30 minutes at 4°C. After adding Quenching Solution to stop biotinylation, the cells were washed and harvested by gentle scraping and lysed using the provided lysis buffer in the presence of a protease inhibitor cocktail (MilliporeSigma). To capture biotinylated proteins, protein lysates were incubated with Neutravidin Agarose gel for 1 hour at room temperature and then washed 5 times. The bound surface proteins were eluted from Neutravidin Agarose by incubation with elution buffer. The eluted plasma membrane proteins were determined by Western blot.

### RAF1 pulldown assay.

RAS activity was assessed using GST-RAF1-RBD beads (catalog RF02, Cytoskeleton) according to the manufacturer’s protocol. Total cell lysate (500 μg) was incubated with GST-RAF1-RBD beads (10 μl) overnight at 4°C with gentle rocking. Samples were washed 5 times and then dissolved in 20 μl 2× SDS sample buffer. Activated HRAS and KRAS were determined by Western blot using HRAS and KRAS antibodies mentioned above, respectively.

### ELISA of soluble NOGOB.

Soluble NOGOB ELISA assay was carried out according to manufacturer’s instruction (catalog 432807, BioLegend). The cells were cultured in 6-well plates with EBM-2 medium supplemented with 20% FBS for 24 hours. Cells were transfected with nonsilencing siControl and siNGBR overnight. Then, the cells were cultured in serum-free medium for 24 hours and the supernatants were collected for measuring soluble NOGOB. Anti-human NOGOB precoated 96-well strip microplates were incubated with the collected medium samples for 2 hours at room temperature. After rinsing with wash buffer, human-soluble NOGOB detection antibody was applied, incubated for 1 hour at room temperature, rinsed in wash buffer, and incubated with Avidin-HRP solution for 30 minutes. Finally, wells were rinsed and incubated with a substrate solution for 15 minutes in the dark. The soluble NOGOB concentration was quantified using the PerkinElmer Multimode Plate Reader to measure absorbance at 450 nm and 570 nm.

### Animal studies.

The male athymic *nu/nu* mice (8 weeks old) were purchased from Jackson Laboratory, and all animal experiments were maintained in the animal facility at the Medical College of Wisconsin. Mice were randomly divided into 2 different groups. All the experiments were carried out with 1.5 × 10^6^ cells per mice. HemSCs were trypsinized, counted, and resuspended in phenol red-free Matrigel (BD Biosciences). The mixture of cells and Matrigel (200 μl/animal) was injected into the backs of 8-week-old male athymic *nu/nu* mice (*n* = 6/group). The animals were monitored every day. Animals were euthanized to harvest Matrigel plugs on day 10 and day 20. The Matrigel plugs were then fixed in 10% neutral buffered formalin for histology analysis. Angiogenesis and adipogenesis were confirmed with H&E staining as well as immunostaining of paraffin-embedded tissue sections.

### Statistics.

Statistical analysis was performed using GraphPad Prism (version 8.0.2) for statistical analysis. The 2-tailed unpaired Student’s *t* test was used to determine *P* values when comparing 2 groups. When comparing multiple groups, 1-way ANOVA was performed with a post hoc Bonferroni’s test to determine which groups showed significant differences. Differences were considered statistically significant when *P* values were less than 0.05.

### Study approval.

All of the animal experiments were approved by the Institutional Animal Care and Use Committee at the Medical College of Wisconsin. Animal care was in accordance with institutional guidelines. Specimens of hemangiomas were obtained from the Division of Pediatric Pathology at the Medical College of Wisconsin with approval from its IRB.

## Author contributions

WH, ZL, ZF, PEN, and SR performed and interpreted the majority of the experiments. QRM and WH conceived the project, designed the experiments, and wrote the manuscript. VS, PEN, SNK, JB, and MMH provided reagents and critical input for the project. QRM supervised the project. All the authors commented on the manuscript.

## Supplementary Material

Supplemental data

Supplemental Table 1

## Version 1 01/05/2021

In-Press Preview

## Version 2 02/08/2021

Electronic publication
