# Supplementary material for: NOGOB receptor–mediated RAS signaling pathway is a target for suppressing proliferating hemangioma
Source: JCI Insight. 2021 Feb 8;6(3):e142299. doi: 10.1172/jci.insight.142299 (PMC7934876; doi:10.1172/jci.insight.142299)
Supplement: Supplemental data [file jciinsight-6-142299-s178.pdf]

Supplemental Figures

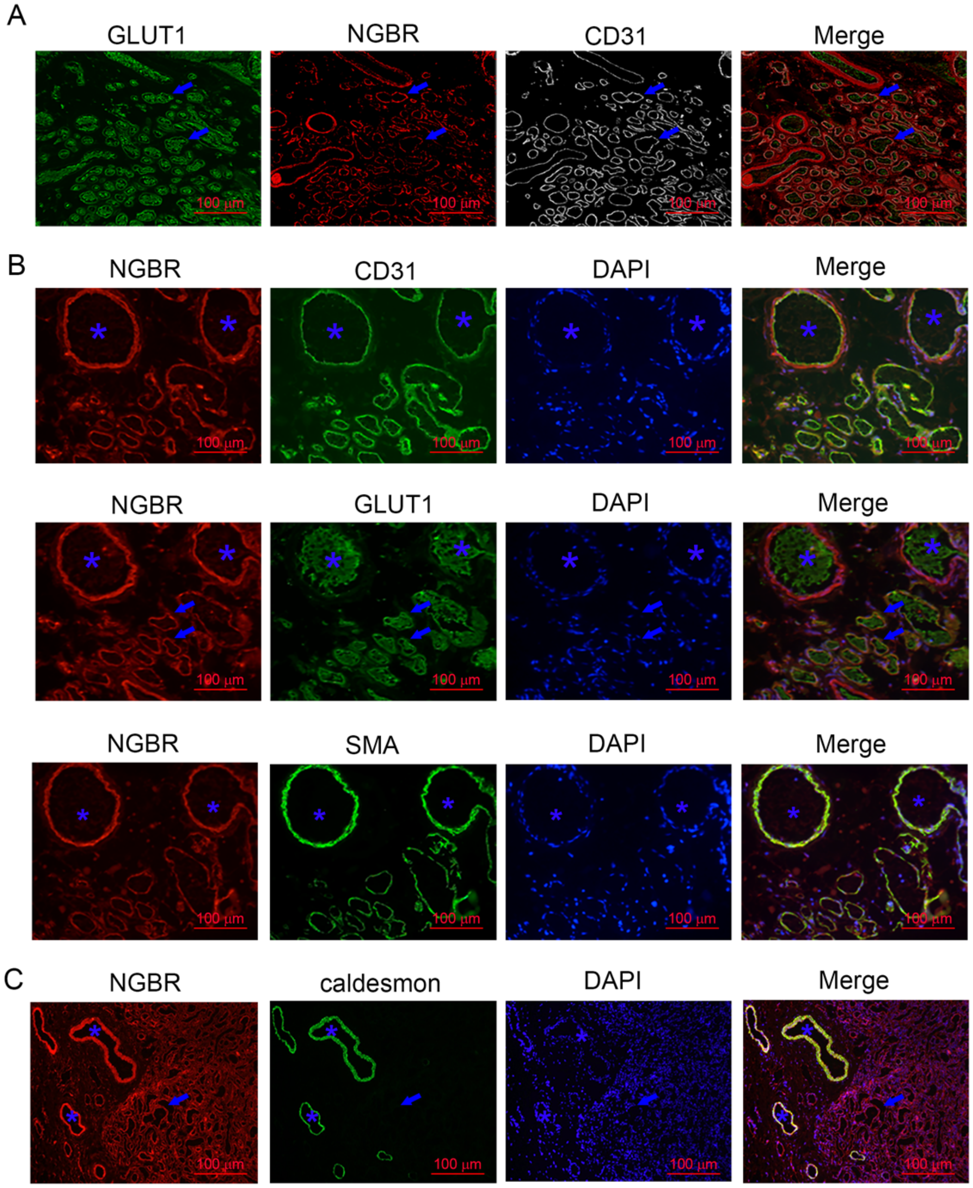

Figure S1. NGBR highly expressed in GLUT1 positive blood vessels in proliferating hemangioma. CD31, GLUT1, NGBR, SMA and caldesmon were used for characterizing hemangioma tissue. (A) Hemangioma sections were immunostained with antibodies against CD31, NGBR and GLUT1. NGBR was detected in GLUT1 positive hemangioma endothelial cells. In all the images, arrows point to the blood vessels showing positive staining of CD31, NGBR, and GLUT1. (B-C) Hemangioma sections were immunostained with anti-CD31, GLUT1, SMA, and caldesmon antibodies for distinguishing hemangioma blood vessels from healthy vasculature. Stars point to the healthy blood vessels and arrows lead to the hemangioma blood vessel. Scale bar: 100 μm.

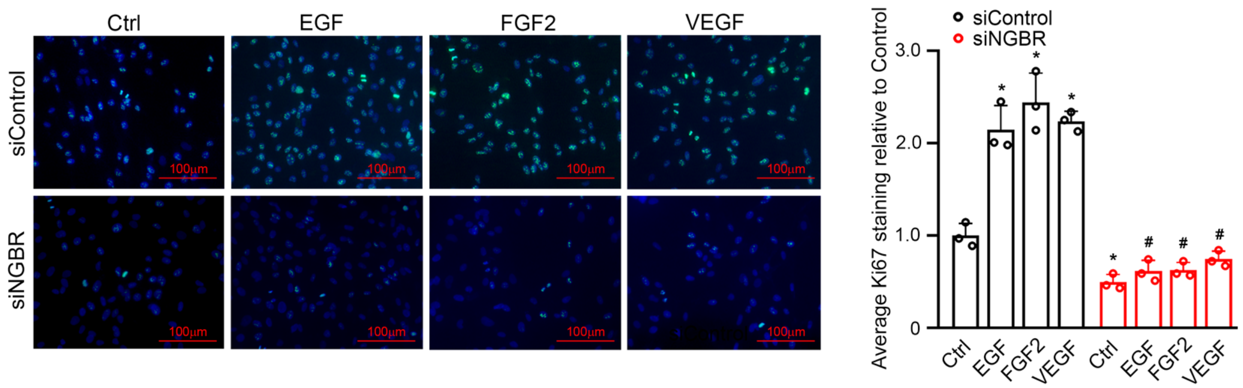

Figure S2. NGBR knockdown reduces the number of Ki67 positive HemSCs. Control HemSCs (siControl) and NGBR-deficient HemSCs (siNGBR) were cultured in EBM-2 medium with 20% FBS for 24 hours, and the Ki67 positive cells were determined by immunofluorescence staining after treatment with EGF/FGF2/VEGF in serum-free medium overnight. Quantitative analysis of positive Ki67 staining was determined by ImageJ. **P*<0.05 versus control (siControl) cells. #*P*<0.05 versus control (siControl) cells treated with EGF/FGF2/VEGF (n=3), 3 repeats. Statistical analyses: 1-way ANOVA with Bonferroni's post hoc test; Data are expressed as the mean ± SEM.

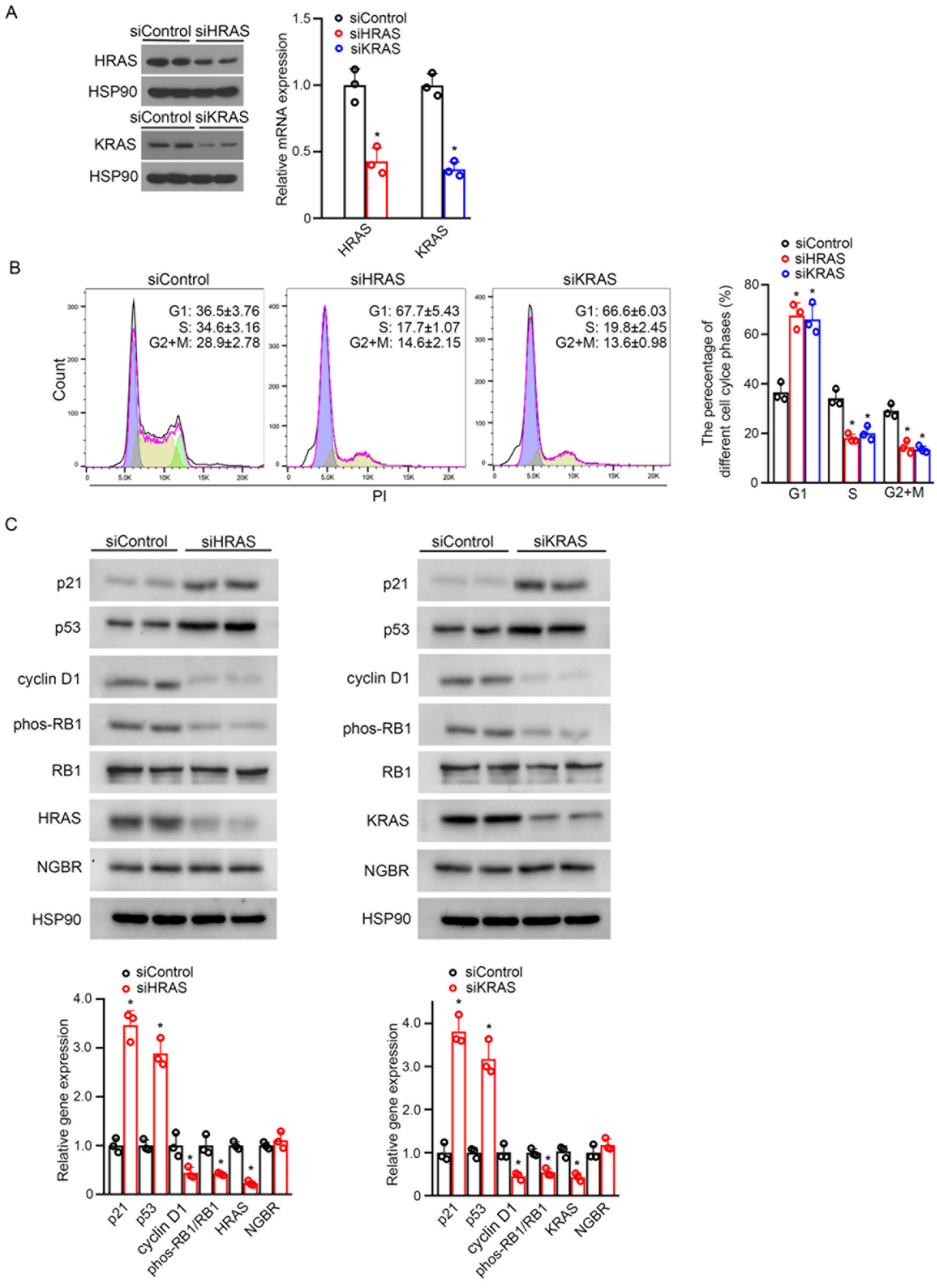
A
siControl siHRAS
HRAS
HSP90
siControl siKRAS
KRAS
HSP90
siControl
siHRAS
siKRAS
Relative mRNA expression
1.5
1.0
0.5
0
HRAS
KRAS
B
siControl
siHRAS
siKRAS
G1: 36.5±3.76
S: 34.6±3.16
G2+M: 28.9±2.78
G1: 67.7±5.43
S: 17.7±1.07
G2+M: 14.6±2.15
G1: 66.6±6.03
S: 19.8±2.45
G2+M: 13.6±0.98
Count
PI
The perecentage of different cell cylce phases (%)
80
60
40
20
0
G1
S
G2+M
C
siControl siHRAS
siControl siKRAS
p21
p53
cyclin D1
phos-RB1
RB1
HRAS
KRAS
NGBR
HSP90
Relative gene expression
4.0
3.0
2.0
1.0
0
p21
p53
cyclin D1
phos-RB1/RB1
HRAS
KRAS
NGBR

Figure S3. The depletion of either HRAS or KRAS in HemSCs causes cell cycle arrest. (A) The efficacy of HRAS or KRAS knockdown with either siHRAS or siKRAS was determined by western blot and real-time reverse-transcription PCR. *$P<0.05$ versus control (siControl) cells (n=3). (B) HemSCs transfected with siHRAS or siKRAS undergo G1 phase arrest (left panel). The cell cycle distribution was analyzed by flow cytometry using propidium iodide (PI) staining. The percentage of individual cell cycle phases presented in the histogram has been summarized in the bar graph (right panel). *$P<0.05$ versus control (siControl) cells (n=3), 3 repeats. (C) Protein levels of NGBR, HRAS, KRAS, p21, p53, cyclin D1, RB1 and phosphorylated RB1 in siControl and siHRAS/siKRAS HemSCs were determined by western blot. Quantitative analysis of phosphorylated proteins was carried out using ImageJ and was normalized to total proteins correspondingly (lower panel). *$P<0.05$ versus control (siControl) cells (n=3). Statistical analyses: 2-tailed unpaired Student’s t test (A, B and C); Data are expressed as the mean ± SEM.

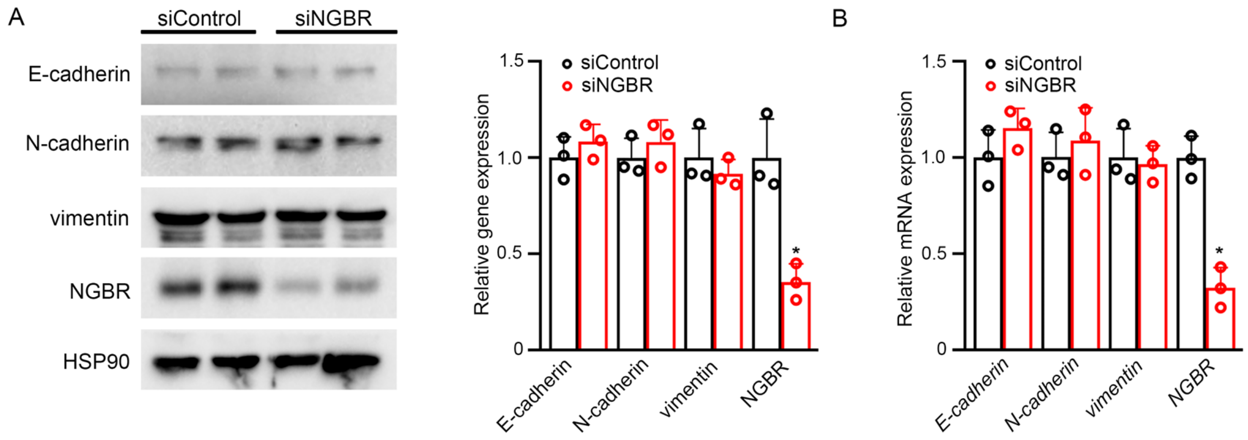

Figure S4. NGBR knockdown does not change the expression levels of mesenchymal markers (vimentin and N-cadherin) and epithelial maker (E-cadherin). (A) Protein levels in siControl and siNGBR-treated HemSCs were determined by western blot. No statistical significance (n=3). (B) Transcriptional levels were determined by real-time reverse-transcription PCR. No statistical significance (n=3). Statistical analyses: 2-tailed unpaired Student's t test (A and B); Data are expressed as the mean ± SEM.

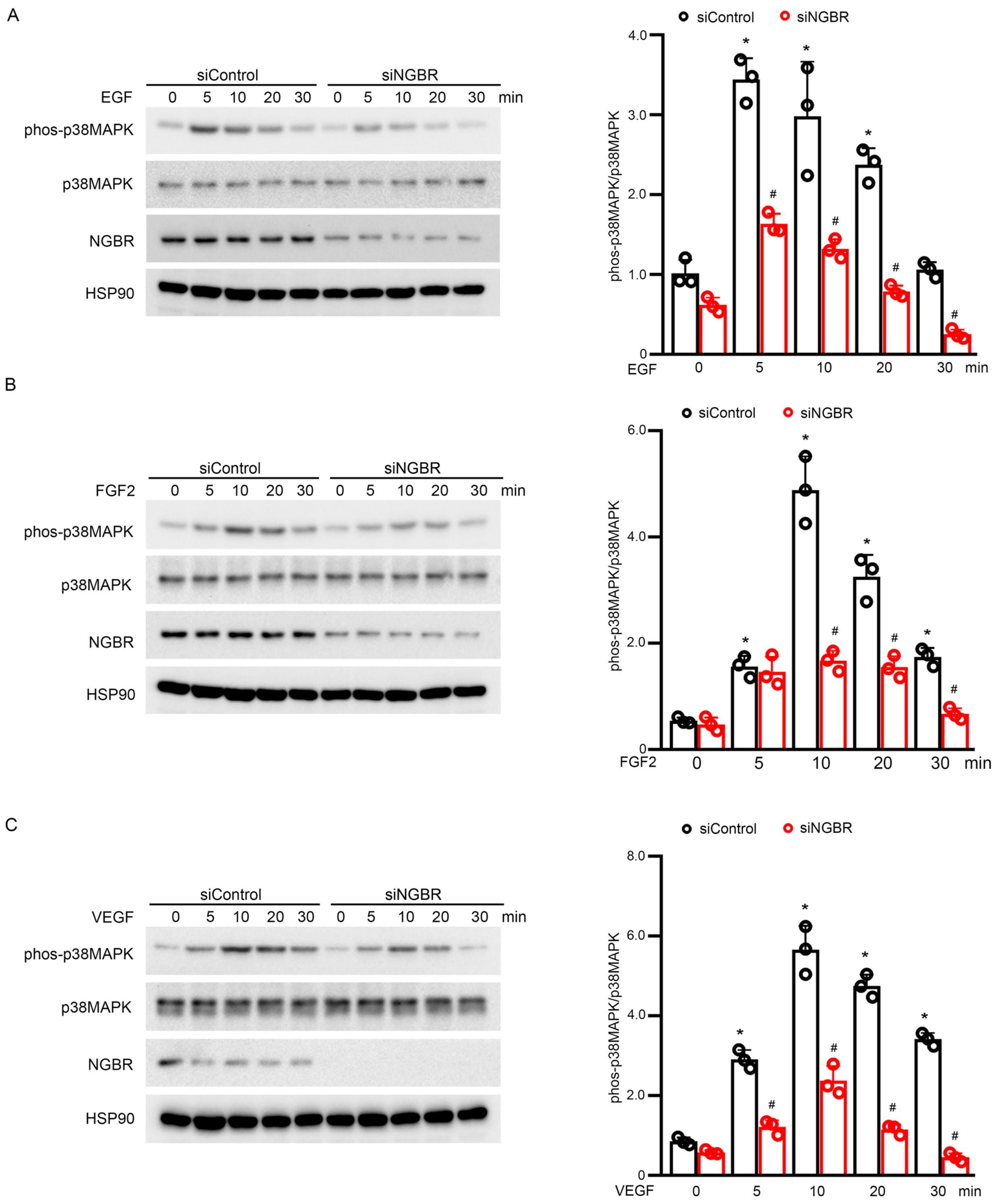

Figure S5. NGBR knockdown attenuates growth factors-induced phosphorylation of p38MAPK in HemSCs. Protein levels were determined by western blot. NGBR knockdown reduces the EGF(A), FGF2 (B), and VEGF(C)-induced phosphorylation of p38MAPK in HemSCs. At 24 h after siControl or siNGBR transfection, cells were arrested overnight in serum-free medium and then stimulated with EGF (100 ng/ml),

FGF2 (100 ng/ml), and VEGF (100 ng/ml) in serum-free medium at indicated times (5, 10, 20, 30 minutes). The growth factor-induced phosphorylation of p38MAPK were determined by western blot. Total p38MAPK, and HSP90 protein levels were used as respective loading controls. Quantitative analysis of phosphorylated proteins was carried out using ImageJ and were normalized to total proteins correspondingly. *P<0.05 versus control (siControl) cells. $^{\#}P$<0.05 versus control (siControl) cells treated by EGF/FGF2/VEGF (n=3). Statistical analyses: 1-way ANOVA with Bonferroni's post hoc test (A, B and C); Data are expressed as the mean ± SEM.

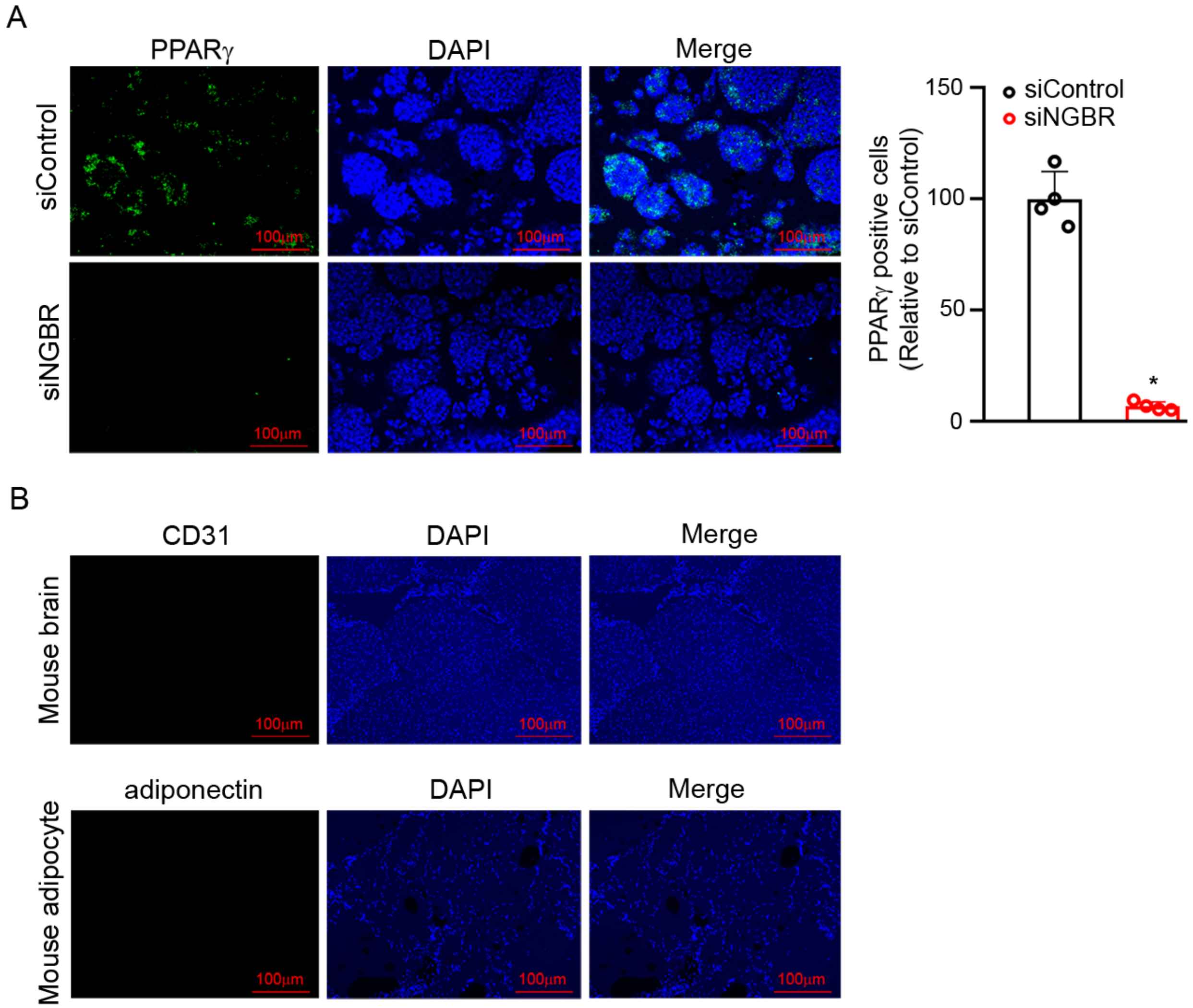

Figure S6. NGBR knockdown suppresses HemSCs differentiation into adipocytes in vivo. (A) Immunofluorescent staining of day 20 implants. Immunostaining of human PPARγ (green) is shown on the left panel, followed by DAPI (blue) staining, and a merged image. NGBR knockdown decreases adipogenesis at the indicated time point. Quantitative analysis of positive PPARγ staining was determined by ImageJ. **P*<0.05 versus control (siControl) (n=4). (B) Negative IF staining signal in the mouse brain or adipocyte tissues confirms the species specificity of anti-human CD31 and adiponectin antibodies. IF staining of human CD31 or human adiponectin is shown on the left panel, followed by DAPI (blue) staining, and a merged image. Statistical analyses: 2-tailed unpaired Student's t test (A); Data are expressed as the mean ± SEM.

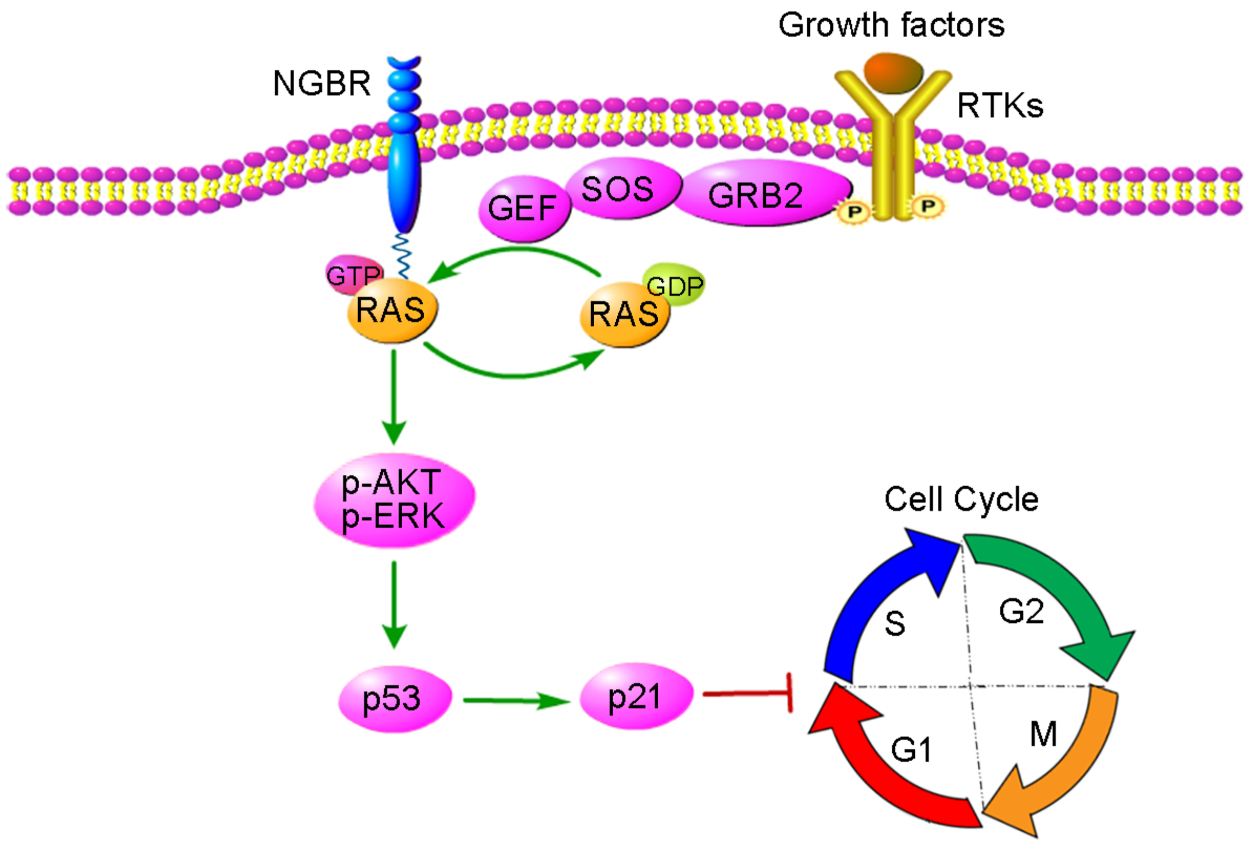

Figure S7. NOGOB receptor is required for receptor tyrosine kinases-induced differentiation of hemangioma stem cells. Growth factors bind to receptor tyrosine kinases (RTKs) and recruit GRB2, an adaptor protein, and SOS, an activator for RAS, to the plasma membrane. NGBR promotes the translocation of RAS to the plasma membrane and enhances RAS downstream signaling, such as phosphorylation of AKT and extracellular signal-related kinase (ERK1/2), which promotes p53 ubiquitination leading to its degradation and diminishes p53-mediated p21-related cell cycle arrest.

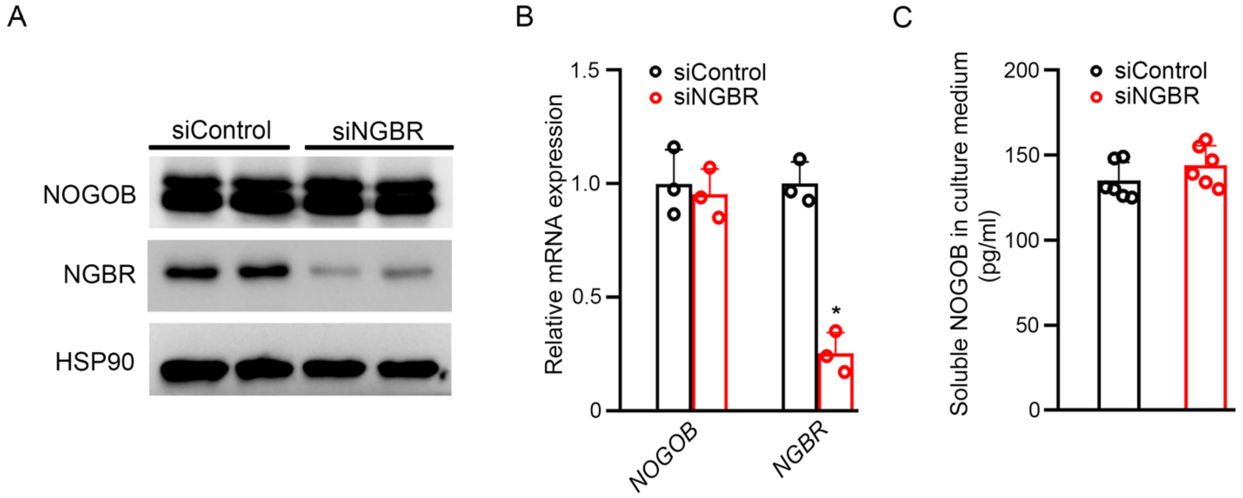

Figure S8. NGBR knockdown does not affect the expression of NOGOB in HemSCs and soluble NOGOB in the culture medium of HemSCs. (A) Protein levels were determined by western blot analysis. (B) Transcriptional levels were determined by real-time reverse-transcription PCR. **P*<0.05 versus control (siControl) cells (n=3). (C) The protein levels of soluble NOGOB in the culture medium of HemSCs treated with siControl or siNGBR were determined by ELISA kit. No statistical significance (n=6). Statistical analyses: 2-tailed unpaired Student's t test (B and C); Data are expressed as the mean ± SEM.

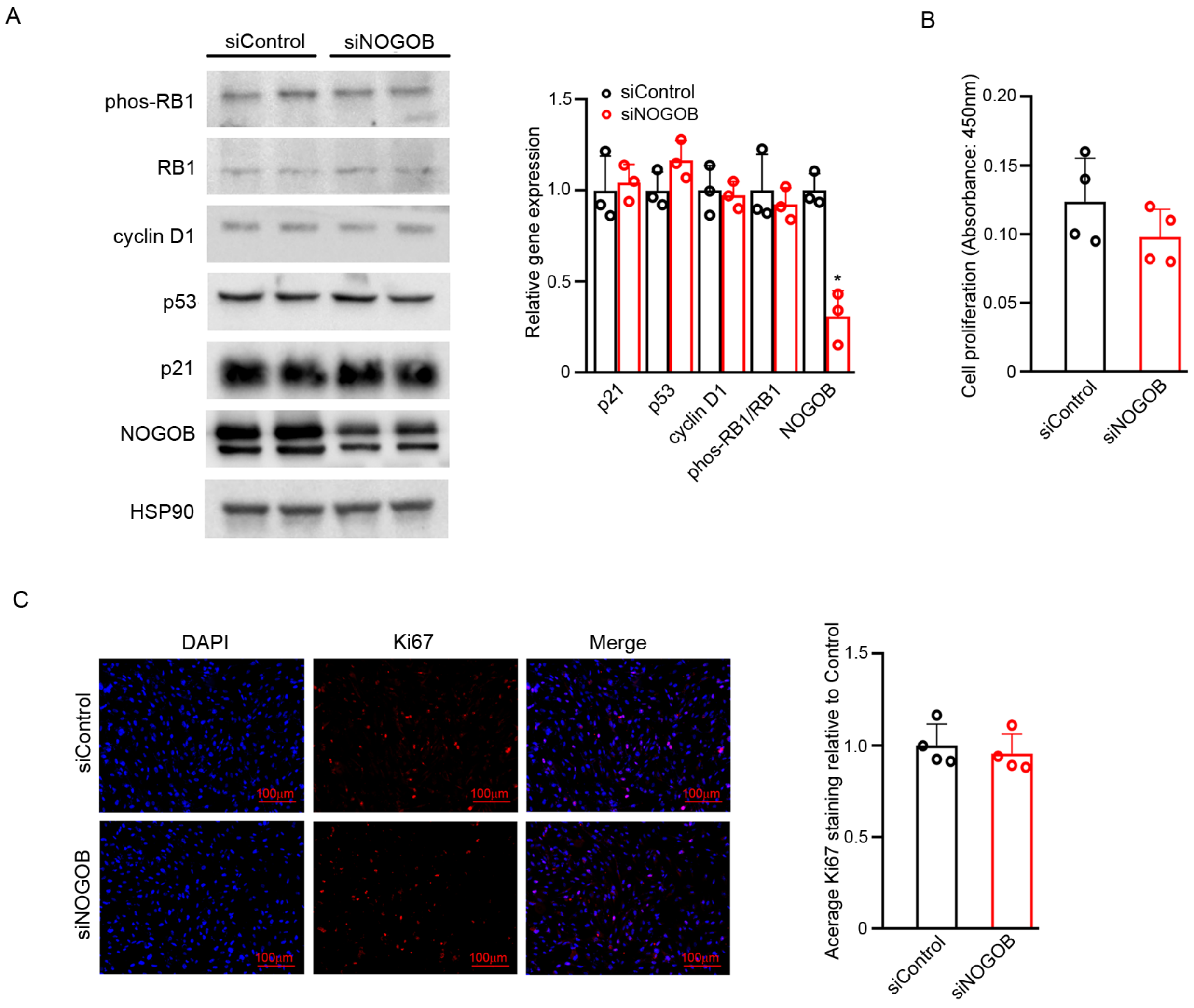

Figure S9. NOGOB knockdown does not affect the proliferation and the expression levels of cell cycle regulators in HemSCs. (A) Protein levels of NOGOB, NGBR, p21, p53, cyclin D1, RB1, and phosphorylated RB1 in siControl and siNOGOB-treated HemSCs were determined by western blot. *$P<0.05$ versus control (siControl) cells (n=3). (B) Cell proliferation modulated by NOGOB deficiency in HemSCs was evaluated by BrdU-based cell proliferation assay kit. The results were expressed as fold change relative to the initial cell number. No statistical significance (n=4), 3 repeats. (C) NOGOB knockdown do not change the number of Ki67 positive HemSCs. Control HemSCs (siControl) and NOGOB-deficient HemSCs (siNOGOB) were cultured in EBM-2 medium with 20% FBS for 24 hours, and the Ki67 positive cells were determined by immunofluorescence staining. Quantitative analysis of positive Ki67 staining was determined by ImageJ. No statistical significance (n=4), 3 repeats. Statistical analyses: 2-tailed unpaired Student's t test (A, B and C); Data are expressed as the mean ± SEM.

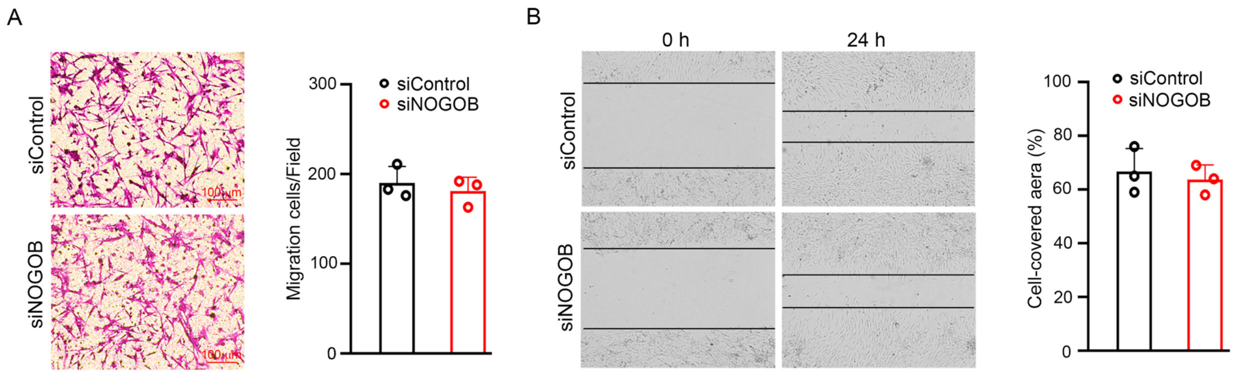

Figure S10. NOGOB knockdown does not affect the migration of HemSCs. (A) Transwell migration assay of HemSCs transfected with siControl, siNOGOB, respectively. Cell migration was determined by using Corning transwell chambers. The results presented are an average of migrated cell numbers in four random microscopic fields from 3 independent samples. (B) Scratch wound healing assay shows no changed migration of HemSCs transfected with siRNA targeting NOGOB. HemSCs transfected with siControl or siNOGOB were subjected to scratch-wound healing assay. The representative images of wound healing are shown at indicated times. The bar graph in the right panel represents the percentage of the cell-covered area determined from each time point. No statistical significance (n=3), 3 repeats. Statistical analyses: 2-tailed unpaired Student's t test (A and B); Data are expressed as the mean ± SEM.
