# Supplementary material for: NOGOB receptor–mediated RAS signaling pathway is a target for suppressing proliferating hemangioma
Source: JCI Insight. 2021 Feb 8;6(3):e142299. doi: 10.1172/jci.insight.142299 (PMC7934876; doi:10.1172/jci.insight.142299)
Supplement: Supplemental Table 1 [file jciinsight-6-142299-s179.pdf]

Supplemental Table S1. Primers for real-time PCR

Gene Name (Species: Human)	Forward Primer	Reverse Primer
NOGOB receptor (**NGBR**)	5'-tgccagttagtagcccagaagcaa-3'	5'-tgatgtgccagggaagaaagccta-3'
vascular endothelial growth factor receptor 2 (**VEGFR2**)	5'-atagaaggtgcccaggaaaag-3'	5'-gtcttcagttcccctccattg-3'
receptor tyrosine-protein kinase erbB-2 (**HER2**)	5'-gagtgtcagccccagaatg-3'	5'-gtaggagaggtcaggtttcac-3'
receptor tyrosine-protein kinase erbB-3 (**HER3**)	5'-acttttctctactggcgtgg-3'	5'-ttccttagctctgtctctttgaag-3'
hematopoietic progenitor cell antigen CD34 (**CD34**)	5'-gtgtctactgctggtcttgg-3'	5'-agggtgttttgggaatagctc-3'
lymphatic vessel Endothelial hyaluronic acid receptor 1(**LYVE1**)	5'-ttagcccaaaccccaagtg-3'	5'-tctggaatgcacgagttagtc-3'
epidermal growth factor receptor (**EGFR**)	5'-aagccatatgacggaatccc-3'	5'-ggaactttgggcgactatctg-3'
fibroblast growth factor receptor 1 (**FGFR1**)	5'-aacctgccttatgtccagatc-3'	5'-agagtccgatagagttacccg-3'
beta-actin (**ACTB**)	5'-ttctacaatgagctgcgtgtggct-3'	5'-tagcacagcctggatagcaacgta-3'
neurite outgrowth Inhibitor B (**NOGOB/RTN4B**)	5'-cgggctcagtggttgttga-3'	5'-actgtcaatgaaagcagcagga-3'
epithelial cadherin (**E-cadherin**)	5'-cccaatacatctcccttcacag-3'	5'-ccacctctaaggccatctttg-3'
neural cadherin (**N-cadherin**)	5'-cccaagacaaagagacccag-3'	5'-gccactgtgcttactgaattg-3'
**vimentin**	5'-cgtgaataccaagacctgctc-3'	5'-ggaaaagtttggaagaggcag-3'
